# Genomic landscape of locally advanced rectal adenocarcinoma: Comparison between before and after neoadjuvant chemoradiation and effects of genetic biomarkers on clinical outcomes and tumor response

**DOI:** 10.1002/cam4.6169

**Published:** 2023-06-01

**Authors:** Tae Hoon Lee, Bum‐Sup Jang, Ji Hyun Chang, Eunji Kim, Jeong Hwan Park, Eui Kyu Chie

**Affiliations:** ^1^ Department of Radiation Oncology Seoul National University Hospital Seoul Republic of Korea; ^2^ Department of Clinical Medical Science Seoul National University College of Medicine Seoul Republic of Korea; ^3^ Department of Radiation Oncology Seoul Metropolitan Government‐Seoul National University Boramae Medical Center Seoul Republic of Korea; ^4^ Department of Pathology Seoul Metropolitan Government‐Seoul National University Boramae Medical Center Seoul Republic of Korea; ^5^ Department of Radiation Oncology Seoul National University College of Medicine Seoul Republic of Korea; ^6^ Medical Research Center, Institute of Radiation Medicine Seoul National University Seoul Republic of Korea

**Keywords:** neoadjuvant chemoradiation, rectal cancer, whole‐exome sequencing

## Abstract

**Purpose:**

To explore genomic biomarkers in rectal cancer by performing whole‐exome sequencing.

**Materials and Methods:**

Pre‐chemoradiation (CRT) biopsy and post‐CRT surgical specimens were obtained from 27 patients undergoing neoadjuvant CRT followed by definitive resection. Exomes were sequenced to a mean coverage of 30×. Somatic single‐nucleotide variants (SNVs) and insertions/deletions (indels) were identified. Tumor mutational burden was defined as the number of SNVs or indels. Mutational signatures were extracted and fitted to COSMIC reference signatures. Tumor heterogeneity was quantified with a mutant‐allele tumor heterogeneity (MATH) score. Genetic biomarkers and frequently occurred copy number alterations (CNAs) were compared between pre‐ and post‐CRT specimens. Their associations with tumor regression grade (TRG) and clinical outcomes were explored.

**Results:**

Top five mutated genes were *APC*, *TP53*, *NF1*, *KRAS*, and *NOTCH1* for pre‐CRT samples and *APC*, *TP53*, *NF1*, *CREBBP*, and *ATM* for post‐CRT samples. Several gene mutations including *RUNX1*, *EGFR*, and *TP53* in pre‐CRT samples showed significant association with clinical outcomes, but not with TRG. However, no such association was found in post‐CRT samples. Discordance of driver mutation status was found between pre‐ and post‐CRT samples. In tumor mutational burden analysis, higher number of SNVs or indels was associated with worse treatment outcomes. Six single‐base substitution (SBS) signatures identified were SBS1, SBS30, SBS29, SBS49, SBS3, and SBS44. The MATH score decreased after CRT on paired analysis. Less than half of CNAs frequent in post‐CRT samples were present in pre‐CRT samples.

**Conclusion:**

Pre‐ and post‐CRT samples showed different genomic landscape. Potential genetic biomarkers of pre‐CRT samples found in the current analysis call for external validation.

## 1 INTRODUCTION

Neoadjuvant chemoradiation (CRT) followed by definitive surgery is a standard of care for patients with locally advanced rectal cancer (LARC). It is well‐known that response after neoadjuvant CRT can significantly impact clinical outcomes.^1^ Several studies have reported that patients with complete response after neoadjuvant therapy may benefit from good rectal preservation and pelvic tumor control with a “Watch‐and‐Wait” strategy.^2^, ^3^ Furthermore, applying more intense CRT for those expected to have a poor response or dismal prognosis prior to treatment initiation might improve overall outcome. Based on these approaches, prediction of response and prognosis prior to neoadjuvant CRT could help tailor individualized treatment. Statically significant models to predict tumor response to CRT based on integrated clinical factors have been reported.^4^ However, no model is widely accepted for clinical practice.

There have been several attempts to utilize genomic features to predict tumor response and prognosis in rectal cancer. Several studies have reported promising results of predicting outcomes based on gene expression profiling, although these studies lack concordance to integrate such results into routine practices.^5^ Recent advance in molecular biology has enabled the sequencing of large amounts of DNA in a short period of time. Such high‐throughput sequencing has been applied to various cancers. Several studies have analyzed whole‐exome sequencing (WES) materials from rectal tumors to predict tumor response and prognosis after neoadjuvant CRT.^6^, ^7^ While each study has shown statistical significance on its own to predict outcomes, inconsistency among studies remains as an obstacle to introduce these models to clinical practices. Therefore, more robust and validated studies are needed to explore clinically useful genetic markers. The purpose of this study was to provide additional genetic data using tissues acquired before and after CRT and find clinically significant biomarkers for predicting prognosis or tumor response by performing WES for materials obtained from patients with LARC who underwent neoadjuvant CRT followed by definitive surgery.

## 2 MATERIALS AND METHODS

### 2.1 Tumor samples

This study was approved by the institutional review board (IRB) of Seoul National University Hospital (approval no. H‐2011‐047‐1171). Tissues analyzed in this study were previously donated to the institutional repository with consents of the patients for further research after pathologic diagnosis. Additional informed consent for this study was waived by the IRB. Formalin‐fixed paraffin‐embedded (FFPE) tissues were obtained from patients with LARC (cT3‐4 or N+ without systemic metastasis) who underwent neoadjuvant CRT followed by definitive surgery from 2008 to 2016. Tumors with complete response in post‐CRT surgical samples were excluded as there was no analyzable post‐CRT tumor tissue in such samples. Normal samples were obtained from unaffected and disease‐free sites of biopsy and surgical samples. Pathologic tumor response was evaluated using three‐point tumor regression grade (TRG) system proposed by Ryan et al.^8^

### 2.2 Sample processing

Two tumor samples and one normal sample were retrieved from pre‐CRT biopsy and post‐CRT surgical specimens of each patient for sequencing. Tumor and normal areas were identified in hematoxylin and eosin stained slides and superimposed to unstained slides. Manual macrodissection was performed for corresponding area using a scalpel. Macrodissected FFPE tissue was digested in a cell lysis solution with proteinase K to extract DNA.

### 2.3 Whole‐exome sequencing

To build standard exome capture libraries, an Agilent SureSelect target enrichment protocol for Illumina paired‐end sequencing library and 1 μg input genomic DNA were used. In all cases, the SureSelect Human All Exon V6 probe (Agilent Technologies, Inc.) set was used. FFPE genomic DNA was sheared using a Covaris LE220 Focused‐ultrasonicator (Covaris LLC). A SureSelect All Exon Capture Library was used for exome capture according to the manufacturer's protocol. Indexed libraries were sequenced to a mean coverage of 30× using a NovaSeq 6000 platform (Illumina, Inc.) by Macrogen Incorporated (Seoul, Republic of Korea).

### 2.4 Single‐nucleotide variant and indel analysis

Generated FASTQ files were processed by Clara Parabricks 3.6.1 (NVIDIA Corporation). Somatic single‐nucleotide variants (SNVs) and insertions/deletions (indels) were identified with Mutect2 of GATK 4.2.4.0. As two tumor samples were obtained from each pre‐CRT biopsy and post‐CRT surgical specimens, joint analysis of two BAM files from the same specimen was performed. Identified SNVs and indels were filtered using FilterMutectCalls with additional condition of minimum variant allele fraction (VAF) of 5% and annotated using Funcotator of GATK 4.2.4.0.

Annotated mutations by Catalog of Somatic Mutations in Cancer (COSMIC) (https://cancer.sanger.ac.uk/cosmic) database v84 were selected for clinical enrichment. Correlation of specific gene mutations with clinical outcomes and TRG measured in post‐CRT specimen was analyzed using Maftools.^9^ SNVs and indels from pre‐ and post‐CRT samples were also compared to find changes caused by neoadjuvant CRT. Shared mutations were defined as SNVs and indels located in the gene mutated in both samples. Percentages of shared mutations between pre‐ and post‐CRT samples from the same patient were measured and correlated with clinical outcomes and TRG.

Mutational signatures were extracted and fitted to COSMIC reference signatures (single‐base substitution [SBS] and doublet‐base substitution [DBS], and indel)^10^ using Sigflow 1.5.^11^ Automatic extraction of signatures using Bayesian variant of non‐negative matrix factorization algorithm was performed. Cosine similarity analysis was applied to extracted signatures and COSMIC reference signatures for fitting. Difference of mutational signatures between pre‐ and post‐CRT samples and correlation of percentages of each mutational signatures with clinical outcomes and TRG were analyzed.

Tumor mutational burden was defined as the number of identified SNVs or indels. Difference of tumor mutational burden between pre‐ and post‐CRT samples and correlation of mutational burden with clinical outcomes and TRG were also analyzed.

Mutant‐allele tumor heterogeneity (MATH) score calculated as 100 × median absolute deviation/median of VAF was used for tumor heterogeneity quantification.^12^ MATH scores were calculated for each sample without joint analysis of two samples from the same tumor specimen. Changes of MATH score caused by neoadjuvant CRT were identified by comparing MATH scores of pre‐ and post‐CRT samples. Effects of MATH score on TRG and clinical outcomes were analyzed. SNVs and indels incorporated in tumor mutational burden, mutational signature, and MATH score analyses were not filtered by COSMIC database.

### 2.5 Copy number analysis

BAM files generated in the previous analysis were processed using Sequenza 2.1.2^13^ for copy number alteration (CNA) detection and cellular/ploidy estimation. CNAs were analyzed per sample without joint analysis. Significantly amplified or deleted regions were identified with GISTIC2^14^ when *q*‐value adjusted by false discovery rate was below 0.1. Frequently occurred CNAs were compared between pre‐ and post‐CRT samples.

### 2.6 Clinical outcomes and statistical analysis

Clinical outcomes analyzed in the current study were locoregional control rate (LRCR), distant metastasis‐free rate (DMFR), progression‐free survival rate (PFSR), and overall survival rate (OSR). An LRCR event was defined as a recurrence within the pelvis including anastomotic site and regional pelvic nodal area. A DMFR event was defined as occurrence of distant metastasis. For PFSR, the event was defined as any recurrence or death, while it was death irrespective of cause for OSR. These events were measured from the date of the completion of radiation therapy. Clinical outcomes at defined time points were calculated using Kaplan–Meier estimator.

Associations of genomic biomarkers with clinical outcomes were calculated using univariate Cox proportional hazards model or log‐rank test. Associations between genomic biomarkers and TRG were calculated using Kruskal–Wallis test or Wilcoxon test. *p*‐Value lower than 0.05 was considered as statistically significant. All statistical analyses were performed using R 4.2.0 (The R Foundation for Statistical Computing).

## 3 RESULTS

### 3.1 Patient characteristics and analyzable samples

Paired set of samples were available from 27 patients, including 18 (66.7%) males and 9 (33.3%) females. The median age at diagnosis was 60 years (range, 31–81 years). Total radiation dose ranged from 50.4 to 54.0 Gy in 1.8 Gy per fractions. Concurrent capecitabine was administered to 14 (51.9%) patients and 5‐FU was given to 13 (48.1%) patients. Sphincter sparing surgery, either low anterior resection or ultralow anterior resection, was performed in 13 (48.1%) and 12 (44.4%) patients, respectively. The remaining two (7.4%) patients underwent abdominoperineal resection. The median follow‐up of patients was 69.7 months (range, 4.9–127.6 months). Ten (37.0%) patients experienced recurrence, with distant metastasis being the most frequent one at nine (33.3%) events. There were two isolated local failures and two locoregional failures. Nine (33.3%) patients succumbed to the disease.

Several samples failed to pass quality check of exome capture. A total of 25 patients were able to be analyzed using pre‐CRT samples. Both pre‐CRT tumor and normal samples were analyzable for 17 patients, whereas pre‐CRT normal samples were not analyzable for 8 patients. For post‐CRT samples, tumor and normal samples from 12 patients were able to be sequenced. Eleven patients had analyzable pairs of pre‐ and post‐CRT tumor samples. One patient had only post‐CRT normal sample that passed that quality check. This patient was excluded from further analysis.

### 3.2 SNVs and indels

**FIGURE 1:**
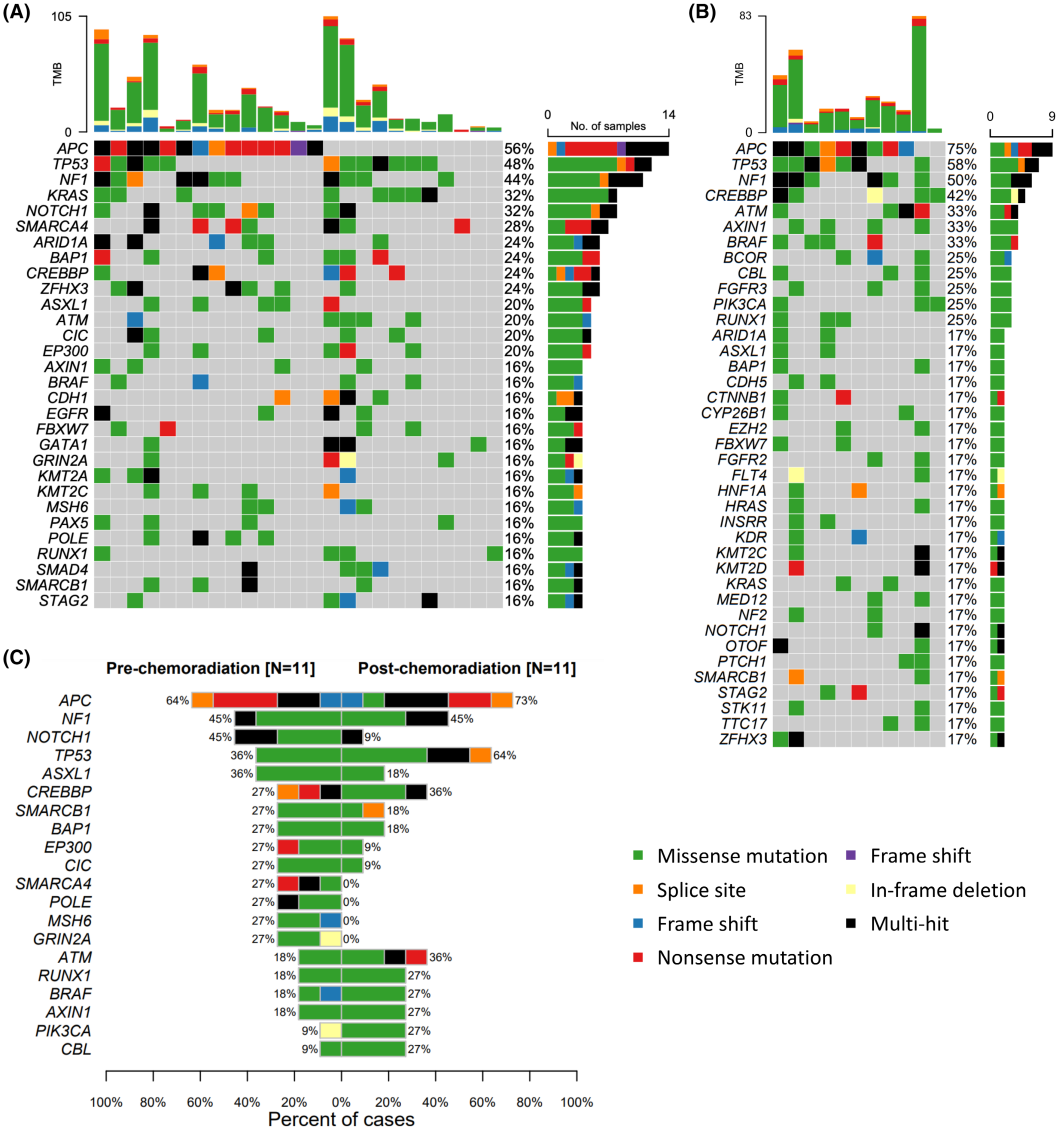
Mutational landscape of (A) pre‐chemoradiation samples and (B) post‐chemoradiation samples. Each column represents a patient and each row represents a gene. Total numbers of identified single‐nucleotide variants and indels of each patient are shown in the top. Mutation rates of specific genes are illustrated in the right. Genes are sorted by mutation rates. (C) Comparison of gene mutation rates between pre‐ and post‐chemoradiation samples in patients with analyzable sample pair. Maftools^9^ package of R 4.2.0 was used for generating the figure.

Pre‐CRT mutational landscape for 25 patients is illustrated in Figure 1A. Genes that mutated in at least four patients were included. Top five frequently mutated genes were *APC*, *TP53*, *NF1*, *KRAS*, and *NOTCH1*. Corresponding genes for colorectal adenocarcinoma data from The Cancer Genome Atlas (TCGA), PanCancer Atlas (https://datacatalog.mskcc.org/dataset/10411, *N* = 594) were *APC*, *TP53*, *TTN*, *KRAS*, and *PIK3CA*. Integrated Genomics Viewer (IGV) screenshots for the top five frequently mutated genes in pre‐CRT samples were illustrated in Figure S1A. *APC*, *TP53*, and *KRAS* were compared with TCGA data as they were major driver mutations of colorectal cancer.^15^ Patients in this study had slightly lower rates of mutations of these genes compared to TCGA data (*APC*, 56.0% vs. 72.5%; *TP53*, 48.0% vs. 58.8%; *KRAS*, 32.0% vs. 40.8%). Other notable genes such as *EGFR*, *BRAF*, *NRAS*, *PIK3CA*, and *SMAD4* were also compared. Patients in this study showed higher mutation rates of *EGFR* (16.0% vs. 2.8%), *BRAF* (16.0% vs. 11.6%), and *SMAD4* (16.0% vs. 12.7%) but a lower mutation rate of *PIK3CA* (12.0% vs. 27.5%) than TCGA data. No *NRAS* and *HRAS* mutations were observed in pre‐CRT samples. Four genes (*MLH1*, *MSH2*, *MSH6*, *PMS2*) associated with microsatellite instability (MSI) were also explored.^16^ There were 2 (8.0%) patients with *MLH1* mutations, 2 (8.0%) patients with *MSH2* mutations, and 4 (16.0%) patients with *MSH6* mutations. No *PSM2* mutation was observed. Six (24.0%) patients had at least one of these MSI‐related gene mutations. Gene mutations in other members of *EGFR* family were observed [*ERBB2*, 2 (8.0%); *ERBB3*, 2 (8.0%); *ERBB4*, 3 (12.0%)]. Co‐mutation plot for pre‐CRT mutations is illustrated in Figure S2A.

Associations of clinical outcomes with specific gene mutations were explored by Cox proportional hazards model. Thirty genes that mutated in at least four patients were analyzed. Genes with significant association with clinical outcomes are summarized in Table 1. Kaplan–Meier curves of clinical outcomes affected by these gene mutations are illustrated in Figure S3. Mutations of MSI genes did not influence clinical outcomes. Relationships of mutations in pre‐CRT samples with TRG were also explored. No gene mutation significantly associated with TRG was discovered.

**TABLE 1:** Mutations in pre‐chemoradiation samples associated with clinical outcomes.

	Hazard ratio	95% confidence interval	*p*‐value
Locoregional control rate			
*SMAD4*	9.571	1.335–68.60	0.025
*EGFR*	7.853	1.077–57.29	0.042
Distant metastasis‐free rate			
*RUNX1*	9.023	2.108–38.62	0.003
Progression‐free survival rate			
*RUNX1*	8.993	2.457–32.91	0.001
*GATA1*	5.019	1.402–17.97	0.013
*BAP1*	3.681	1.119–12.11	0.032
*KMT2A*	4.045	1.047–15.63	0.043
*TP53*	3.971	1.046–15.07	0.043
Overall survival rate			
*RUNX1*	14.64	3.118–68.72	0.001
*APC*	0.187	0.039–0.907	0.038
*EGFR*	4.591	1.075–19.61	0.038
*STAG2*	4.528	1.064–19.27	0.041
*TP53*	4.878	1.008–23.59	0.049

*Note*: Cox proportional hazards model was used for calculation.

Post‐CRT mutational landscape for 12 patients is illustrated in Figure 1B. Genes showing mutations in at least two patients are included in the figure. One patient had none of the 39 frequently mutated genes. Thus, this patient was not included in the figure. Top five frequently mutated genes were *APC*, *TP53*, *NF1*, *CREBBP*, and *ATM*. IGV screenshots for the top five frequently mutated genes in post‐CRT samples were illustrated in Figure S1B. Top three genes remained, whereas mutation rate of *KRAS* and *NOTCH1* decreased from 32.0% to 16.7%. No *NRAS* mutation was observed, but there were two (1.67%) patients with *HRAS* mutation. Among four MSI genes explored, two (16.7%) patients harbored *MLH1* mutations. *MSH2*, *MSH6*, and *PSM2* showed no mutations. There was one (8.3%) patient with *EGFR* mutation and one (8.3%) patient with *ERBB4* mutation. No *ERBB2* and *ERBB3* mutations were observed. Co‐mutation plot for pre‐CRT mutations was illustrated in Figure S2B. Association of clinical outcomes and specific gene mutations were explored for 12 genes showing mutations in at least three patients. Clinical outcomes showed no statistically significant associations with specific gene mutations. When relationships between mutations in post‐CRT samples and TRG were explored, no significant association was found.

### 3.3 Comparison of mutations between pre‐ and post‐CRT samples

Comparison of gene mutations between pre‐ and post‐CRT samples was performed for 11 analyzable patients. There were 20 genes that mutated in at least three patients in pre‐ or post‐CRT samples. There were no statistically significant differences in mutation rates of these genes. Comparison of mutation rates of these genes is illustrated in Figure 1C. Concordance of driver mutations such as *APC*, *TP53*, and *KRAS* between pre‐ and post‐CRT samples was examined. Four (36.4%) patients retained *TP53* status. Two (18.2%) patients had *TP53* mutation present in pre‐CRT samples but not in post‐CRT samples. *TP53* mutation appeared in post‐CRT samples of 5 (45.5%) patients, which was not present in pre‐CRT samples. *KRAS* status was maintained in 9 (81.8%) patients. Two (18.2%) patients gained *KRAS* mutation in post‐CRT samples. Four (36.4%) patients had retained *APC* status. Three (27.3%) patients lost *APC* mutation, whereas four (36.4%) patients gained *APC* mutation.

**FIGURE 2:**
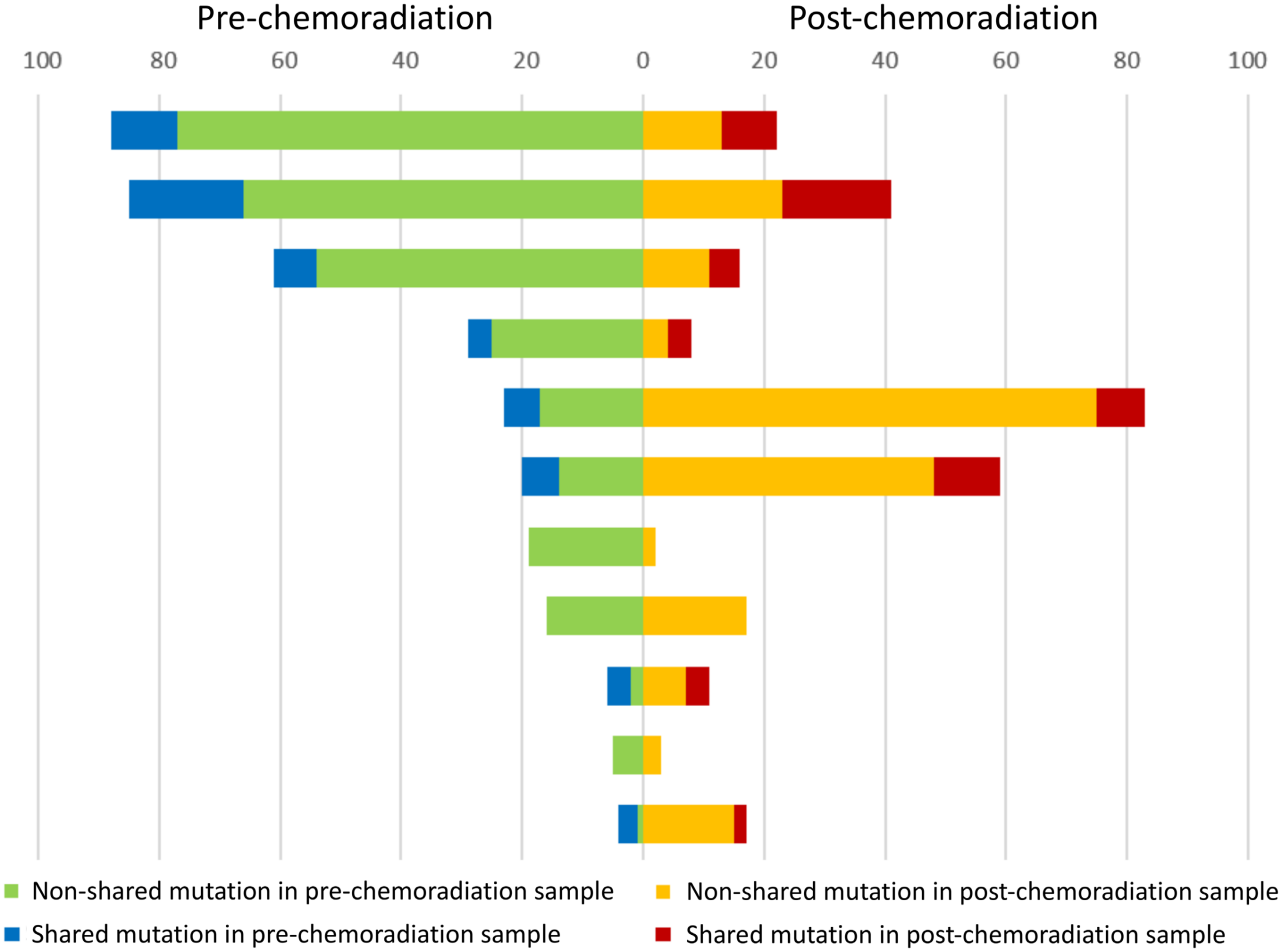
Numbers of shared and non‐shared mutations in pre‐ and post‐chemoradiation samples. Each row represents a patient.

Number of shared mutations that presented in the same gene in both pre‐ and post‐CRT samples was searched for 11 analyzable patients (Figure 2). The median rate of shared mutation among pre‐CRT samples and post‐CRT samples was 13.8% (range, 0.0%–75.0%) and 18.6% (range, 0.0%–50.0%), respectively. There was no significant association between clinical outcomes or TRG and rate of shared mutation.

### 3.4 Mutational signature

**FIGURE 3:**
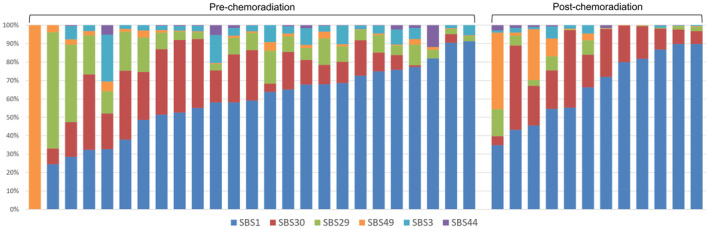
Relative compositions of single‐base substitution signatures in pre‐ and post‐chemoradiation samples sorted by rates of SBS1. Each column represents a patient.

Relative compositions of SBS mutational signatures for each patient are illustrated in Figure 3. Six COSMIC SBS signatures were identified: SBS1 (spontaneous deamination of 5‐methylcytosine), SBS30 (defective DNA base excision repair due to NTHL1 mutations), SBS29 (tobacco chewing), SBS49 (possible sequencing artifact), SBS3 (defective homologous recombination DNA damage repair), and SBS44 (defective DNA mismatch repair). When mutational signatures of pre‐ and post‐CRT samples for 11 analyzable patients were compared, the relative rate of SBS29 was significantly decreased after CRT from an average of 9.91%–3.92% (paired Wilcoxon singed rank test *p* = 0.024). When associations of clinical outcomes with pre‐CRT mutational signatures were explored, a high SBS30 rate was associated with a worse DMFR (Cox proportional hazards model [hazard ratio, HR]: 1.070 per 1%, 95% confidence interval [CI]: 1.014–1.129, *p* = 0.014) and PFSR (HR: 1.065 per 1%, 95% CI: 1.015–1.117, *p* = 0.011). There were no significant associations between clinical outcomes and post‐CRT mutational signatures. TRG showed no significant association with pre‐ or post‐CRT mutational signatures. Mutation of MSI gene was not associated with relative rate of SBS44 in pre‐ or post‐CRT samples.

Two COSMIC DBS signatures were identified, namely DBS1 (ultraviolet light exposure) and DBS10 (defective DNA mismatch repair). Two COSMIC indel signatures were identified: ID6 (defective homologous recombination DNA damage repair) and ID2 (slippage during DNA replication of the template DNA strand). There were no significant differences in relative rates of DBS and indel signatures between pre‐ and post‐CRT samples. Relative rate of DBS and indel signatures did not impact clinical outcomes. DBS signatures and pre‐CRT indel signatures did not show any significant relationship with TRG. However, in post‐CRT samples, lower ID6 composition was observed in TRG 1 (average relative rate: TRG 1, 87.3%; TRG 2, 100.0%; TRG 3, 97.8%; Kruskal–Wallis test *p* = 0.016). Relative compositions of DBS and indel signatures for each patient are illustrated in Figure S4.

### 3.5 Tumor mutational burden

**FIGURE 4:**
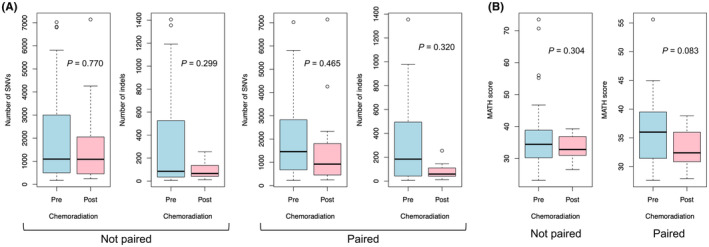
(A) Box plots for numbers of single‐nucleotide variants and indels. (B) Box plots for MATH scores in pre‐ and post‐chemoradiation samples.

The median number of SNVs in pre‐CRT samples without COSMIC database filtering was 1094 (range, 173–7024). The median number of indels in pre‐CRT samples was 85 (range, 6–1406). The median number of SNVs and indels in post‐CRT samples was 1086 (range, 245–7140) and 66 (range, 12–255), respectively. When all pre‐ and post‐CRT samples were compared, no significant difference in the number of SNVs (Wilcoxon rank sum test *p* = 0.770) or indels (*p* = 0.299) was observed. When paired pre‐ and post‐CRT analyzable samples were compared within 11 patients, there was no significant difference in the number of SNVs (paired Wilcoxon signed rank test *p* = 0.465) or indels (*p* = 0.320). Box plots of mutational burden by pre‐ and post‐CRT are illustrated in Figure 4A. When correlations for numbers of SNVs or indels between pre‐ and post‐CRT samples were explored in 11 analyzable patients, no statistically significant correlation was found with assumption of a linear relationship between pre‐ and post‐CRT numbers (SNV, Pearson's correlation coefficient 0.050, *p* = 0.883; indel, Pearson's correlation coefficient −0.139, *p* = 0.684). Scatter plots by numbers of SNVs or indels between pre‐ and post‐CRT samples are illustrated in Figure S5A,B.

Tumor mutational burden of pre‐CRT samples was associated with clinical outcomes. Higher number of SNVs was associated with worse PFSR (HR: 1.029 per 100 SNVs, 95% CI: 1.004–1.054, *p* = 0.024), whereas higher number of indels was associated with worse DMFR (HR: 1.142 per 100 indels, 95% CI: 1.009–1.294, *p* = 0.036) and PFSR (HR: 1.155, 95% CI: 1.035–1.289, *p* = 0.010). There were no significant relationships between tumor mutational burden of post‐CRT samples and clinical outcomes. Associations between tumor mutational burden and clinical outcomes are summarized in Table S1. No significant relationship between TRG and mutational burden was observed. Box plots of mutational burden by TRG are illustrated in Figure S5C,D.

### 3.6 MATH score

Tumor heterogeneity is quantified by MATH score for each sample without joint analysis of two samples from the same biopsy or surgical specimens. The median MATH score for pre‐CRT samples (*N* = 50) and post‐CRT samples (*N* = 24) was 34.43 (range, 23.17–73.50) and 32.83 (range, 26.55–39.60), respectively. When all pre‐ and post‐CRT samples were compared, no difference in MATH score was observed (Wilcoxon rank sum test *p* = 0.304). Paired match was carried out for 11 patients with both sequenced pre‐ and post‐CRT samples. Average MATH score of two tumor samples for each pre‐ and post‐CRT per patient was used for this analysis. MATH score decreased with marginal significance after CRT (average: 37.06 vs. 32.90, paired Wilcoxon signed rank test *p* = 0.083). Box plots of MATH score by pre‐ and post‐CRT are illustrated in Figure 4B. No relationship between pre‐ and post‐CRT MATH scores was observed (*p* = 0.686). No significant associations between clinical outcomes or TRG and MATH score of pre‐ or post‐CRT samples were found. Box plots by TRG and scatter plots by pre‐ and post‐CRT of MATH score are illustrated in Figure S6.

### 3.7 Copy number alteration

**FIGURE 5:**
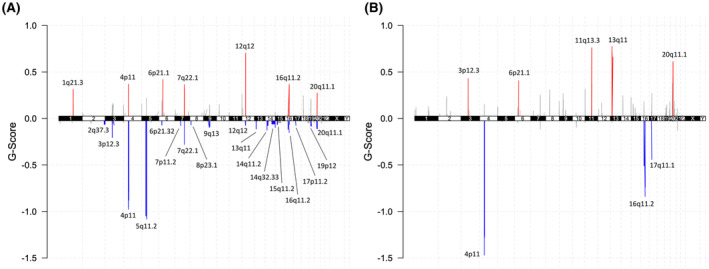
Copy number alterations frequently found in (A) pre‐ and (B) post‐chemoradiation tumor samples. Maftools^9^ package of R 4.2.0 was used for generating the figure.

CNA analysis was performed for 34 pre‐CRT samples of 17 patients and 24 post‐CRT samples of 12 patients as normal pair was required for Sequenza analysis. Estimated sample purity and ploidy with Sequenza is summarized in Table S2. CNAs frequently found in pre‐ and post‐CRT tumor samples are illustrated in Figure 5. The full list of these CNAs is summarized in Table S3. Seven amplification peaks and 21 deletion peaks were found to be significant in pre‐CRT samples. In post‐CRT samples, five amplification peaks and four deletion peaks were significantly altered. Among nine CNAs in post‐CRT samples, four CNAs overlapped with CNA peaks in pre‐CRT samples.

## 4 DISCUSSION

This study explored WES data from pre‐CRT biopsy and post‐CRT surgical specimens of patients with LARC. Noticeable changes in somatic SNVs, indels, genetic biomarkers derived from mutations, and CNAs between pre‐ and post‐CRT samples were observed. Several somatic mutations and genetic biomarkers potentially affecting clinical outcomes were found.

This study found several somatic gene mutations in pre‐CRT associated with clinical outcomes. The strongest association was observed in *RUNX1* mutation. In this study, *RUNX1* mutation was associated with worse DMFR, PFSR, and OSR. *RUNX1* mutation has been mainly studied in hematologic malignancy.^17^ Recent studies have reported that *RUNX1* expression is also prevalent in solid tumor.^18^ Several mechanisms for function of *RUNX1* as an oncogene or a tumor suppressor have been proposed.^19^ Previous reports have analyzed the role of *RUNX1* in colorectal cancer and concluded that *RUNX1* may facilitate cancer cell migration.^20^, ^21^ In this study, *RUNX1* mutation impacted DMFR but not LRCR. It might indicate that worse PFSR and OSR of patients with *RUNX1* mutation were due to more frequent distant metastasis affected by *RUNX1* activity.

In this study, *EGFR* mutation was associated with worse LRCR and OSR. In pre‐CRT samples of this study, 16% of analyzable patients had *EGFR* mutation, though *EGFR* mutations are uncommon in LARC. This high percentage of mutation might be due to the small sample size of this study. However, it might also be due to racial difference as a previous Korean study reported *EGFR* mutation rate of 22.41% in 58 colorectal cancer patients.^22^ Impact of *EGFR* mutation on prognosis of colorectal cancer is questionable. Reports of *EGFR* mutation are scarce due to its rareness, although several studies have explored the association between *EGFR* expression and prognosis.^23^ Contrary to the current analysis, the aforementioned Korean study concluded that there was no association between *EGFR* mutation status and OSR.^22^ Validation with a larger cohort of specific ethnicity may clarify this difference.

*TP53* mutation was also associated with PFSR and OSR in this study. It is well‐known that *TP53* has a role in carcinogenesis of colorectal adenocarcinoma.^24^ Furthermore, mutational analysis of prospective series has revealed that *TP53* mutation is associated with worse PFSR,^25^ concordant with the current finding. *SMAD4* mutation was associated with worse LRCR in this study. It has been reported that loss of *SMAD4* is involved in colorectal carcinogenesis.^26^ Worse prognosis in patients with *SMAD4* mutation has been previously reported.^27^

Four relatively rare gene mutations (*GATA1*, *BAP1*, *KMT2A*, and *STAG2*) were significantly associated with worse PFSR or OSR in this study. GATA1 was considered to play a role in the hematopoietic system.^28^ It might also play a role in colorectal cancer progression, as *GATA1* expression may affect prognosis of colorectal cancer.^29^ *BAP1* mutation is prevalent in other cancer types such as uveal melanoma and malignant mesothelioma. Its prognostic significance in colorectal cancer has also been reported.^30^ *KMT2A* is responsible for histone methyltransferase. The mechanism of its impact on colorectal cancer development has been proposed.^31^ *STAG2* encodes a cohesion subunit. It has been proposed that its mutational inactivation might lead to aneuploidy by dysfunctional chromosomal segregation.^32^ However, impact of *STAG2* mutation in colorectal cancer remains questionable.^33^ Proposed prognostic impact of these uncommon mutations requires independent external validation.

*APC* mutation is one of the most frequent mutations in colorectal adenocarcinoma. It is also one of well‐known driver mutations. In this study, *APC* mutation was associated with better OSR, concordant with previous literature.^34^

No gene mutations in post‐CRT samples impacted clinical outcomes. This was presumably due to the low number of analyzable post‐CRT samples compared to pre‐CRT samples. Statistically significant relationship between specific gene mutations and TRG was not found either.

Several differences of genomic landscape between pre‐ and post‐CRT samples were found in this study. Although differences in mutation rates of the same genes between paired pre‐ and post‐CRT samples were not significant, not all mutations observed in pre‐CRT samples were retained in post‐CRT samples, including driver mutations such as *APC*, *TP53*, and *KRAS*. Yang et al. have compared genomic landscape of 28 pairs of LARC samples between before and after neoadjuvant CRT.^35^ Similar to this study, analyzed tumors were non‐responders to CRT and several genomic differences between pre‐ and post‐CRT samples were found. Toomey et al. have used a similar approach with different conclusion.^36^ WES was performed for pre‐treatment biopsy specimen, on‐treatment biopsy specimen, and surgical tissues. There was no newly found driver mutation when WES results of pre‐treatment biopsy, on‐treatment biopsy, and surgical specimen were compared, although variant allele fractions might differ. Kamran et al. have also compared pre‐ and post‐CRT mutations and reported that gene mutation status occurring frequently in colorectal cancer such as *TP53*, *KRAS*, and *APC* is grossly similar between pre‐ and post‐CRT samples.^37^ Mutational differences between pre‐ and post‐CRT samples reported in this study and the study by Yang et al. might be affected by intra‐tumoral heterogeneity as different tissues obtained in the same rectal tumor might harbor different mutations.^38^ Discordant results between this study and aforementioned studies might also indicate that analyzing mutations in rectal cancer and effect of CRT on mutations is susceptible to difference in detailed methodology of respective studies such as sequencing depth and ways to obtain tissues due to substantial intra‐tumoral heterogeneity of rectal cancer. Additional sequencing data with deeper depth might be one way to address this issue.

Six COSMIC SBS signatures were observed in WES materials of this study. SBS1 and SBS44 are known to be relatively frequent in colorectal adenocarcinoma, but not SBS30, SBS29, or SBS3.^10^ Current findings on mutational signature changes between pre‐ and post‐CRT samples and association between mutational signature and clinical outcomes involve SBS29 and SBS30. Although studies on other primaries have shown significant relationships between certain mutational signatures and clinical outcomes,^39^, ^40^ it is quite difficult to interpret current findings for mutational signatures due to unknown mechanisms behind rare mutational signatures in colorectal adenocarcinoma. Further studies may help to clarify this. This is also true for DBS and indel signatures reported in this study.

Effects of tumor mutational burden on clinical outcomes and TRG were explored in this study. Previous studies with larger cohort have reported that higher tumor mutational burden is associated with better prognosis in colorectal cancer.^41^, ^42^ However, the relationship of tumor mutational burden with clinical outcomes in this study showed contrasting results, with higher numbers of SNVs and indels in pre‐CRT samples showing significant associations with worse PFSR. Limited size of the analyzed cohort might have led to such discordant results.

Tumor heterogeneity was quantified by MATH score in this study. MATH score decreased with marginal statistical significance after CRT in patients with paired pre‐ and post‐CRT samples, whereas clinical outcomes and TRG did not show significant associations with MATH score. In contrast, increase of MATH score after CRT,^43^ associations of MATH score with higher disease stage^43^ and pathologic response^44^ have been reported in the previous literatures. This discordance might be due to the limited size of the cohort. However, decreased MATH score after CRT might be possible if clonal selection by CRT is dominant. The effect of CRT on tumor heterogeneity might be different depending on clinical situations such as response.

This study has several limitations. First, methodology of this study may not have been in concordance with pathogenic variant detection clinically utilized in the current practice. Determination of *RAS* and *BRAF* mutations and *HER2* amplifications by next‐generation sequencing panels was integrated in the clinical guideline,^45^ but such procedures may not be well‐reflected in this study. Mutations found in this study may lack clinical significance as recent database for clinical significance of mutations was not integrated. This may significantly hinder clinical implication of findings from current study. Second, the cohort size of this study might be too small for reliability. Thus, further clarification by independent external validation is needed. Third, the sequencing depth of an average of ×30 was relatively low. Considering substantial heterogeneity of rectal cancer, this might have obscured clonal variations of mutations. In addition, tissues used for the analysis were not optimal. Several tissues did not pass quality check. Contrary to several studies, blood samples, which were used as normal references in other studies, were not obtainable. Despite these limitations, this study showed the genomic landscape of Korean patients with LARC before and after CRT and generated hypotheses for further analysis.

In conclusion, differences of genomic landscape between pre‐ and post‐CRT samples were observed. Several potential genetic biomarkers were found from pre‐CRT samples for prognosis prediction, although independent external validation is needed in the future.

## AUTHOR CONTRIBUTIONS

**Tae Hoon Lee:** Formal analysis (equal); investigation (equal); writing – original draft (equal); writing – review and editing (equal). **Bum‐Sup Jang:** Conceptualization (equal); methodology (equal); writing – review and editing (equal). **Ji Hyun Chang:** Data curation (equal); supervision (equal); writing – review and editing (equal). **Eunji Kim:** Investigation (equal); writing – review and editing (equal). **Jeong Hwan Park:** Investigation (equal); writing – review and editing (equal). **Eui Kyu Chie:** Conceptualization (equal); project administration (equal); supervision (equal); writing – review and editing (equal).

## FUNDING INFORMATION

This work was funded by the National Research Foundation of Korea (NRF) grants funded by the Ministry of Science and ICT, Republic of Korea (NRF‐2020R1F1A1073616) and Patient‐Centered Clinical Research Coordinating Center (PACEN) funded by the Ministry of Health & Welfare, Republic of Korea (HC21C0149) for Prof. Eui Kyu CHIE, and Seoul National University Hospital, Grant Number: 0320210410 for Prof. Ji Hyun CHANG.

## Supporting information


Figure S1.

Figure S2.

Figure S3.

Figure S4.

Figure S5.

Figure S6.

Table S1.

Table S2.

Table S3.

## Data Availability

The data that support the findings of this study are available from the corresponding author upon reasonable request after institutional review board approval.
