# Supplementary material for: Genomic landscape of locally advanced rectal adenocarcinoma: Comparison between before and after neoadjuvant chemoradiation and effects of genetic biomarkers on clinical outcomes and tumor response
Source: Cancer Med. 2023 Jun 1;12(14):15664–75. doi: 10.1002/cam4.6169 (PMC10417181; doi:10.1002/cam4.6169)

Supplementary Figure 1. Integrative Genomics Viewer screenshots for the top five frequently mutated genes in (A) pre- and (B) post-chemoradiation samples.

(A) Pre-chemoradiation samples

APC

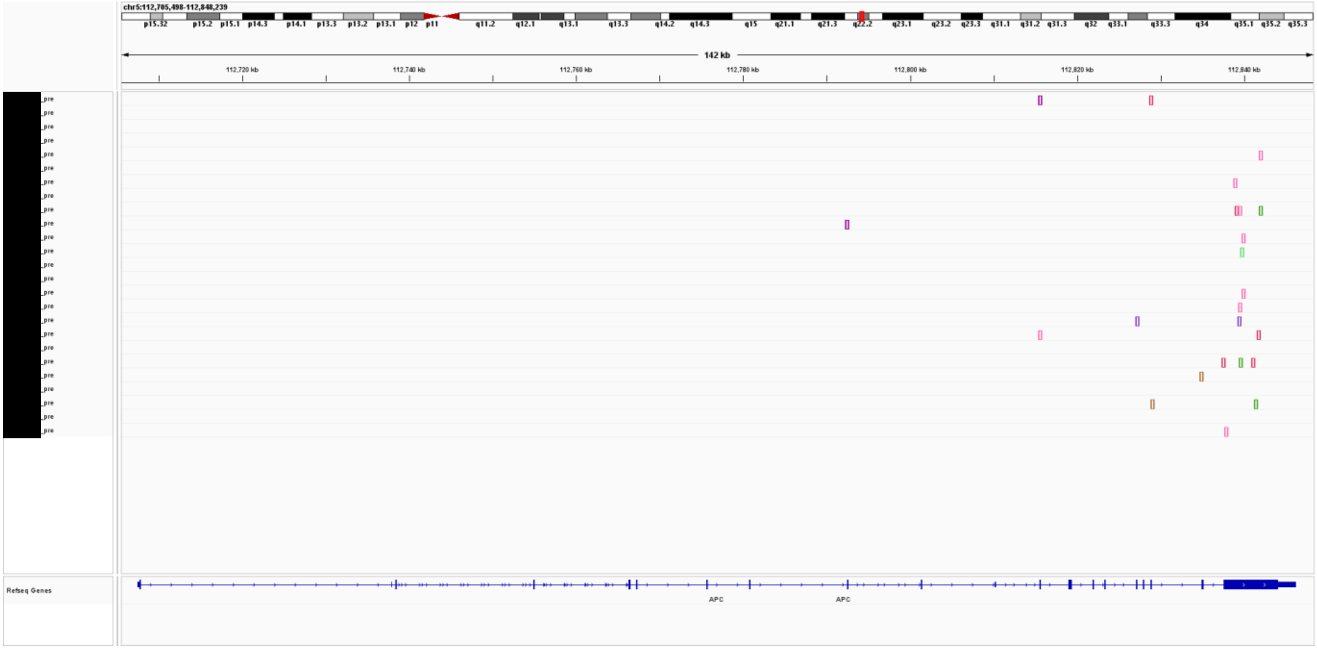

TP53

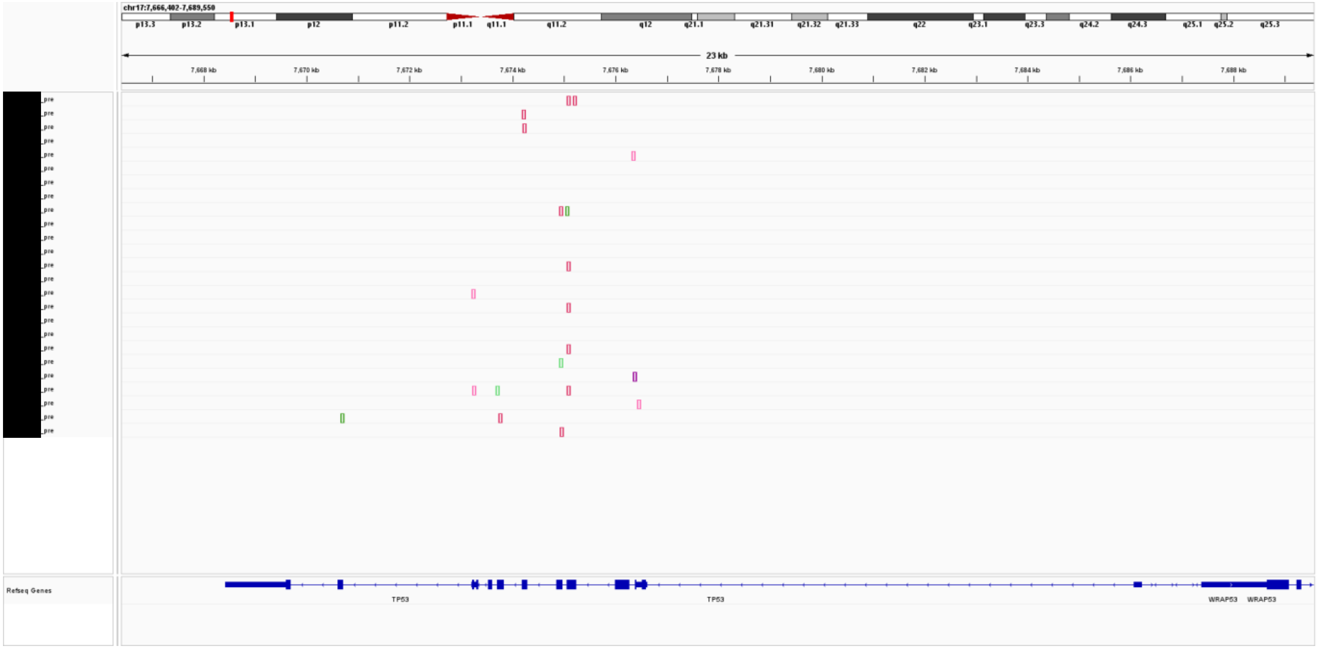

NF1

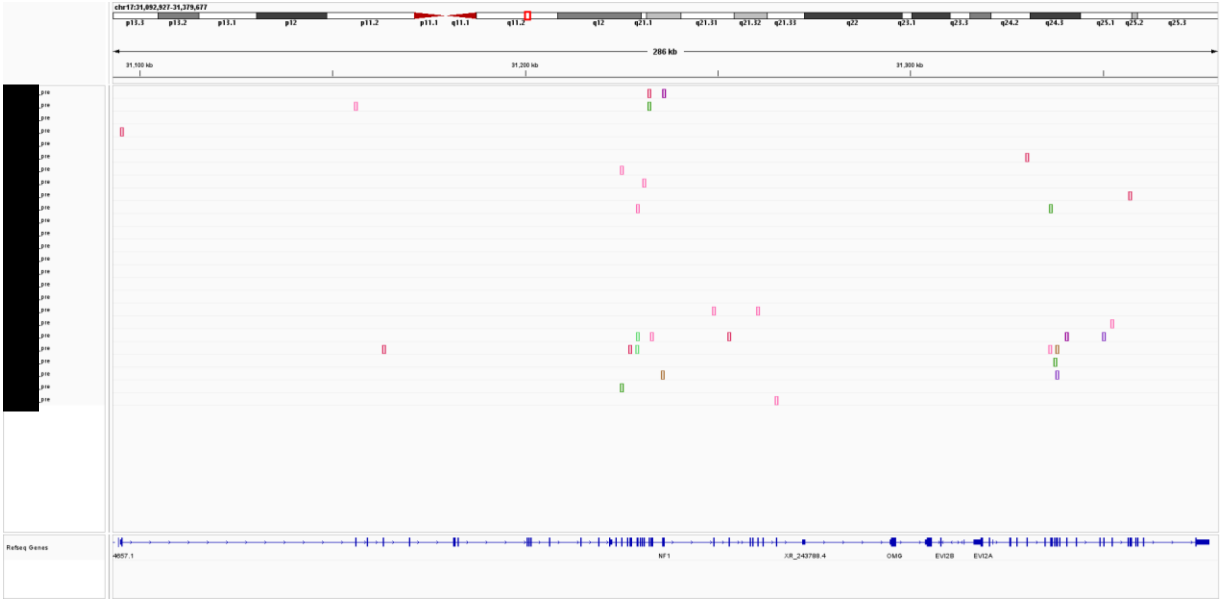

KRAS

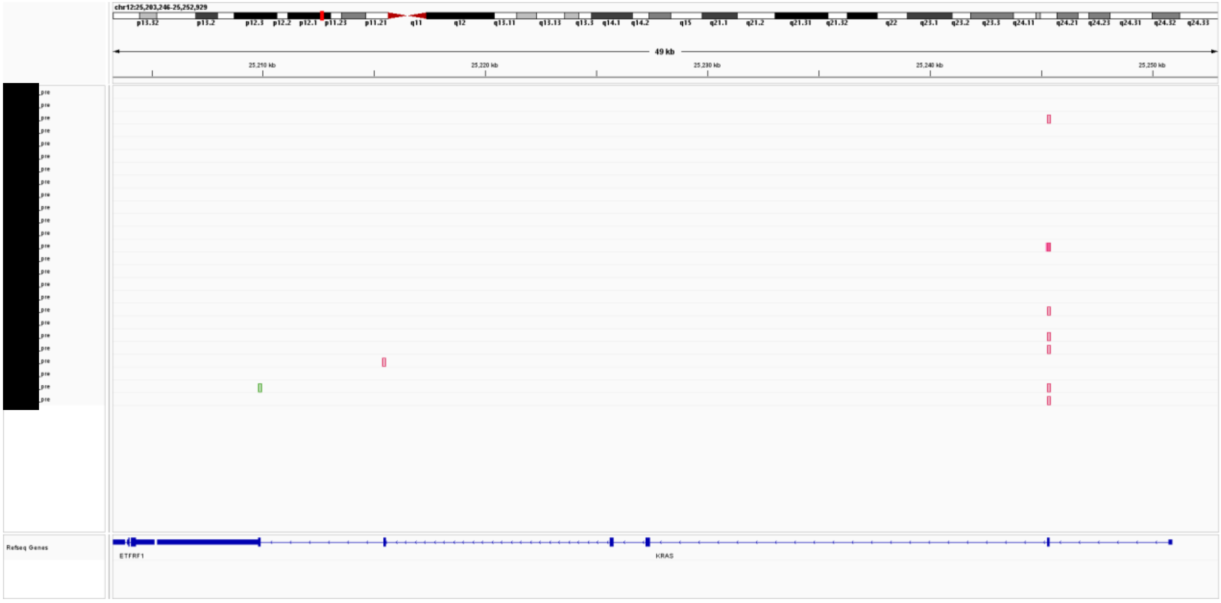

NOTCH1

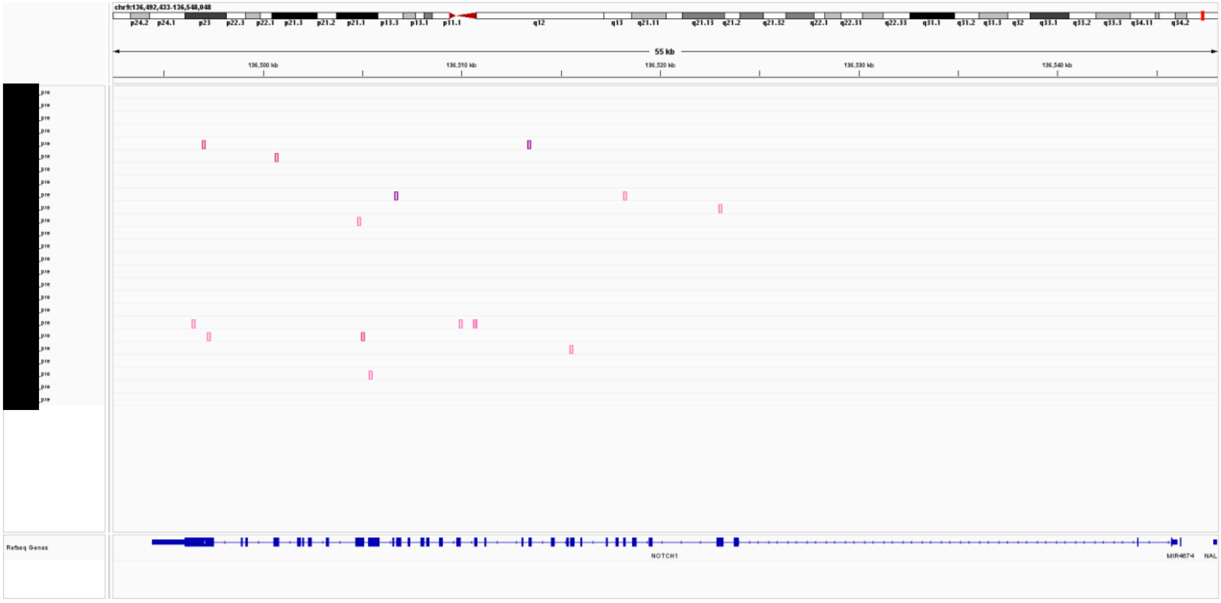

(B) Post-chemoradiation samples

APC

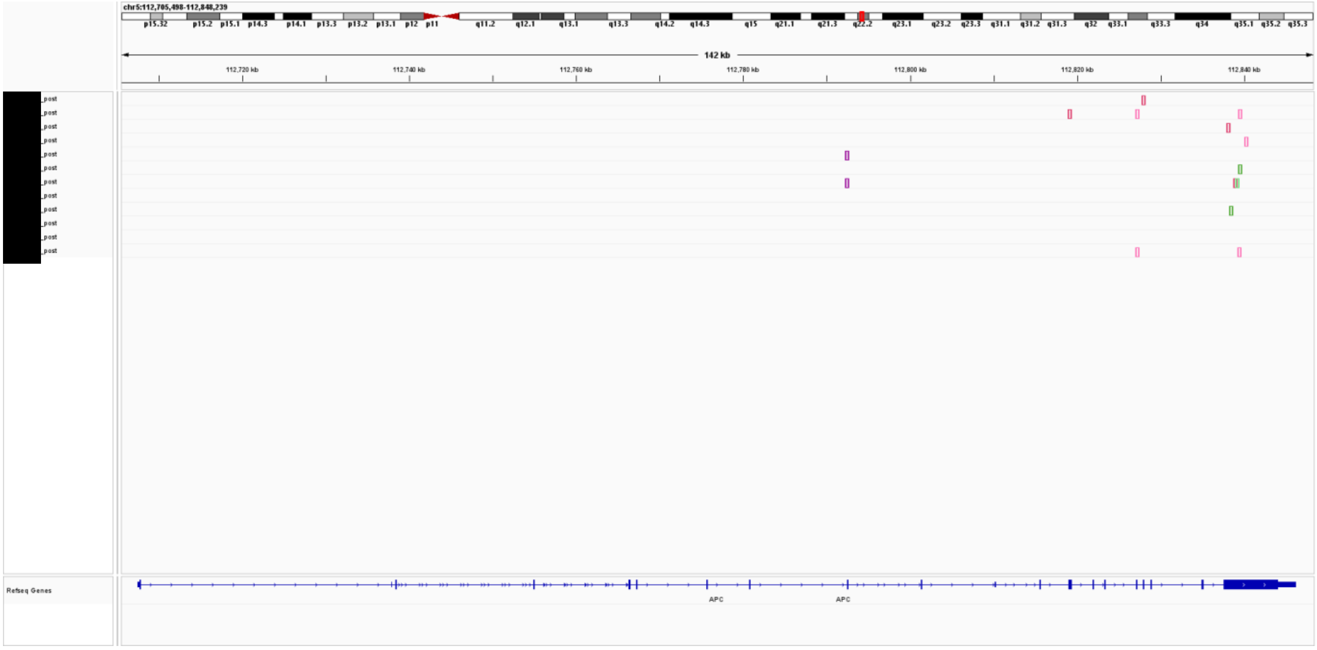

TP53

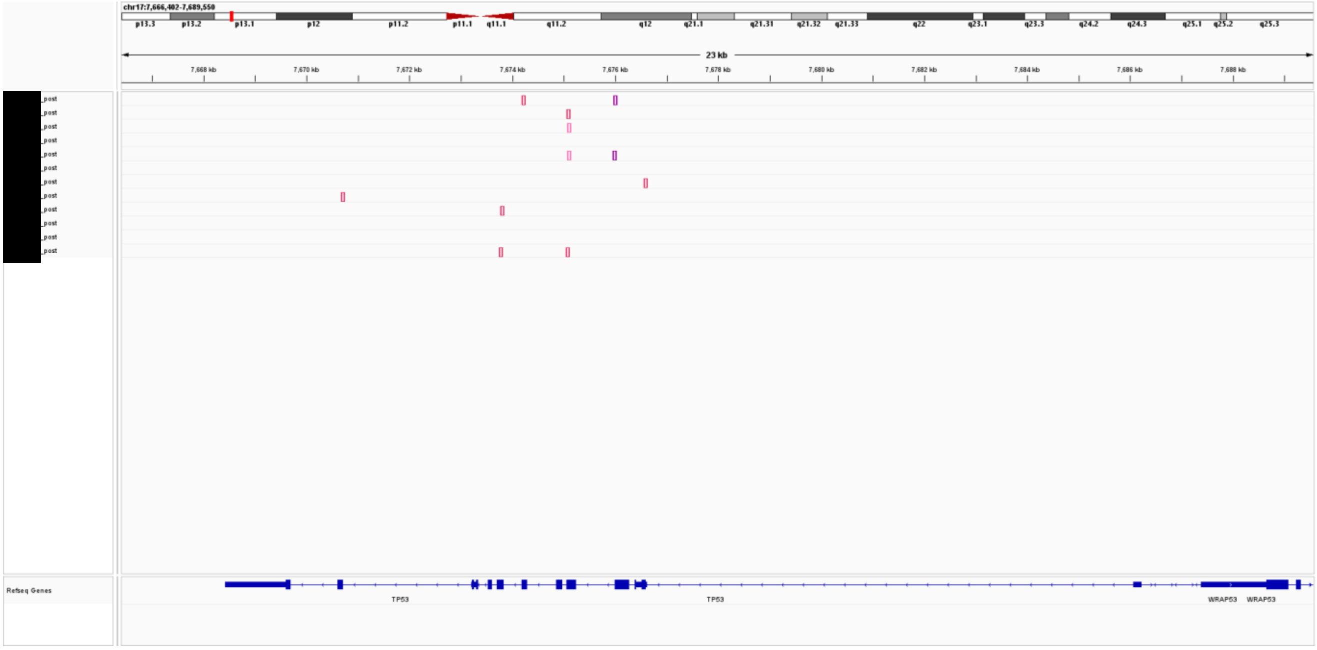

NF1

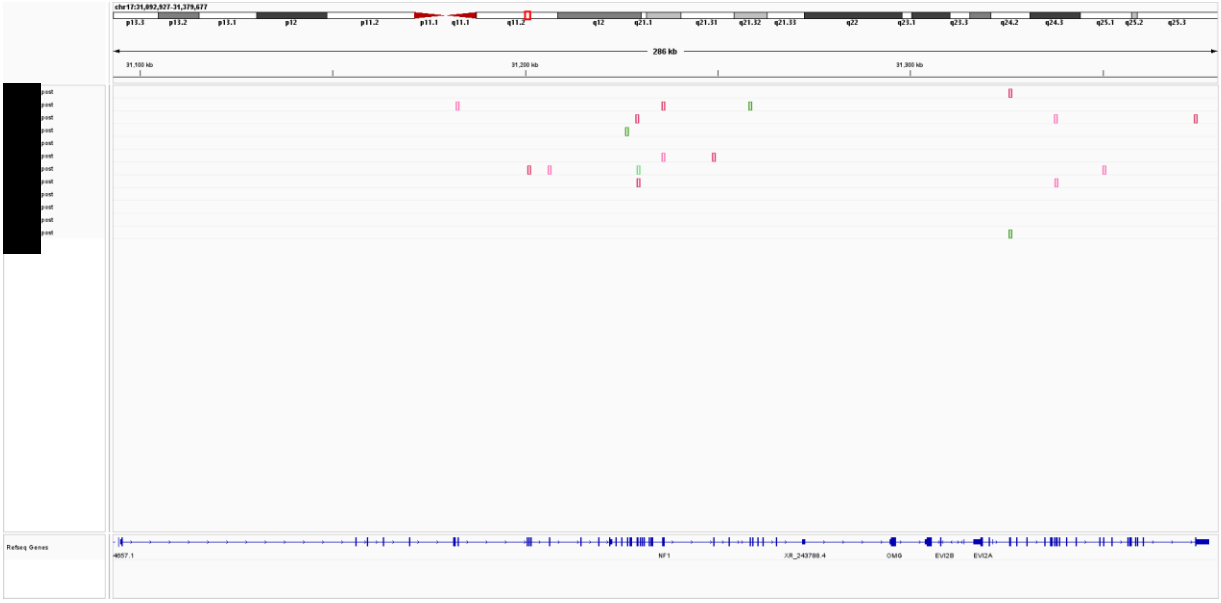

CREBBP

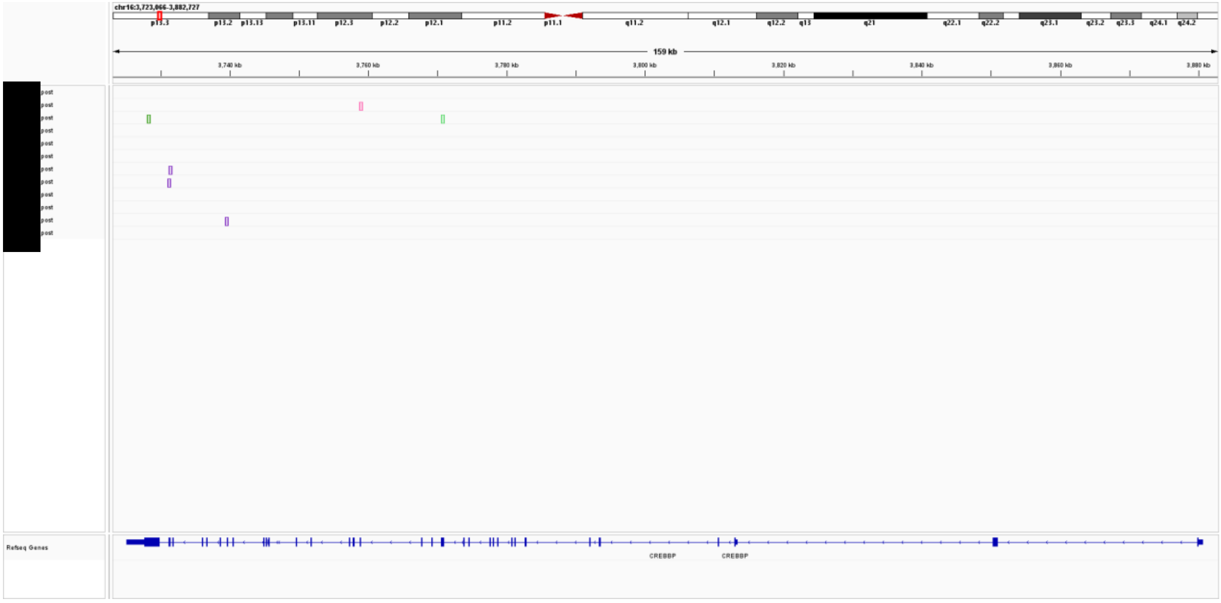

ATM

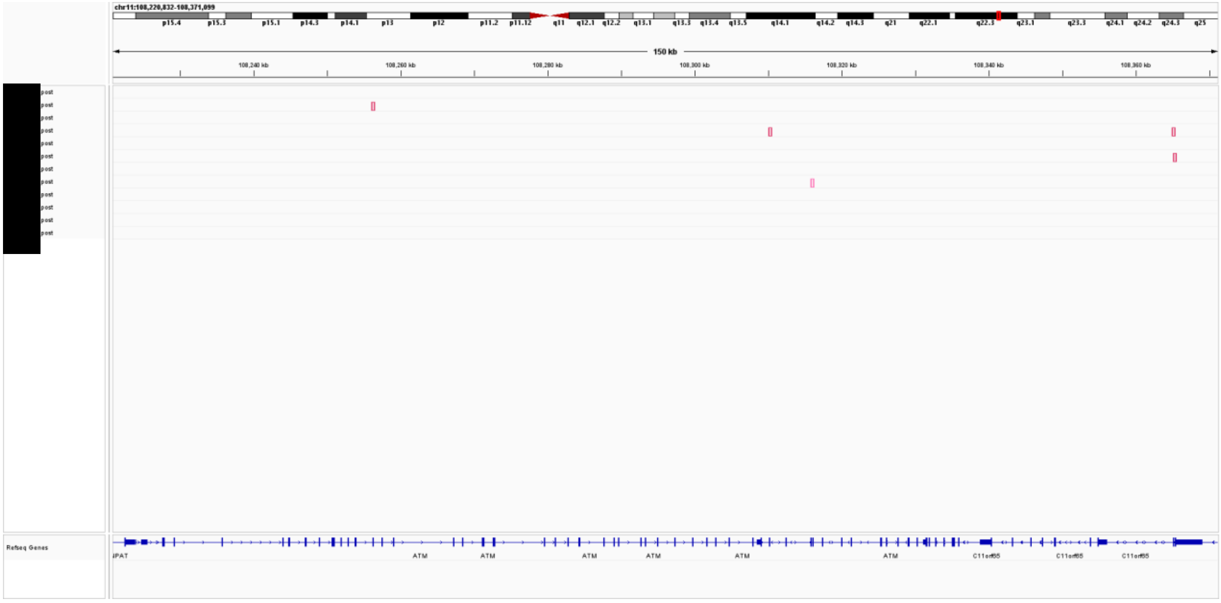

Supplement: Supplementary file 1 — Figure S1. [file CAM4-12-15664-s006.pdf]
