# Supplementary material for: Genomic landscape of locally advanced rectal adenocarcinoma: Comparison between before and after neoadjuvant chemoradiation and effects of genetic biomarkers on clinical outcomes and tumor response
Source: Cancer Med. 2023 Jun 1;12(14):15664–75. doi: 10.1002/cam4.6169 (PMC10417181; doi:10.1002/cam4.6169)

Supplementary Figure 2. Co-mutation plots for gene mutations detected in (A) pre- and (B) post-chemoradiation samples

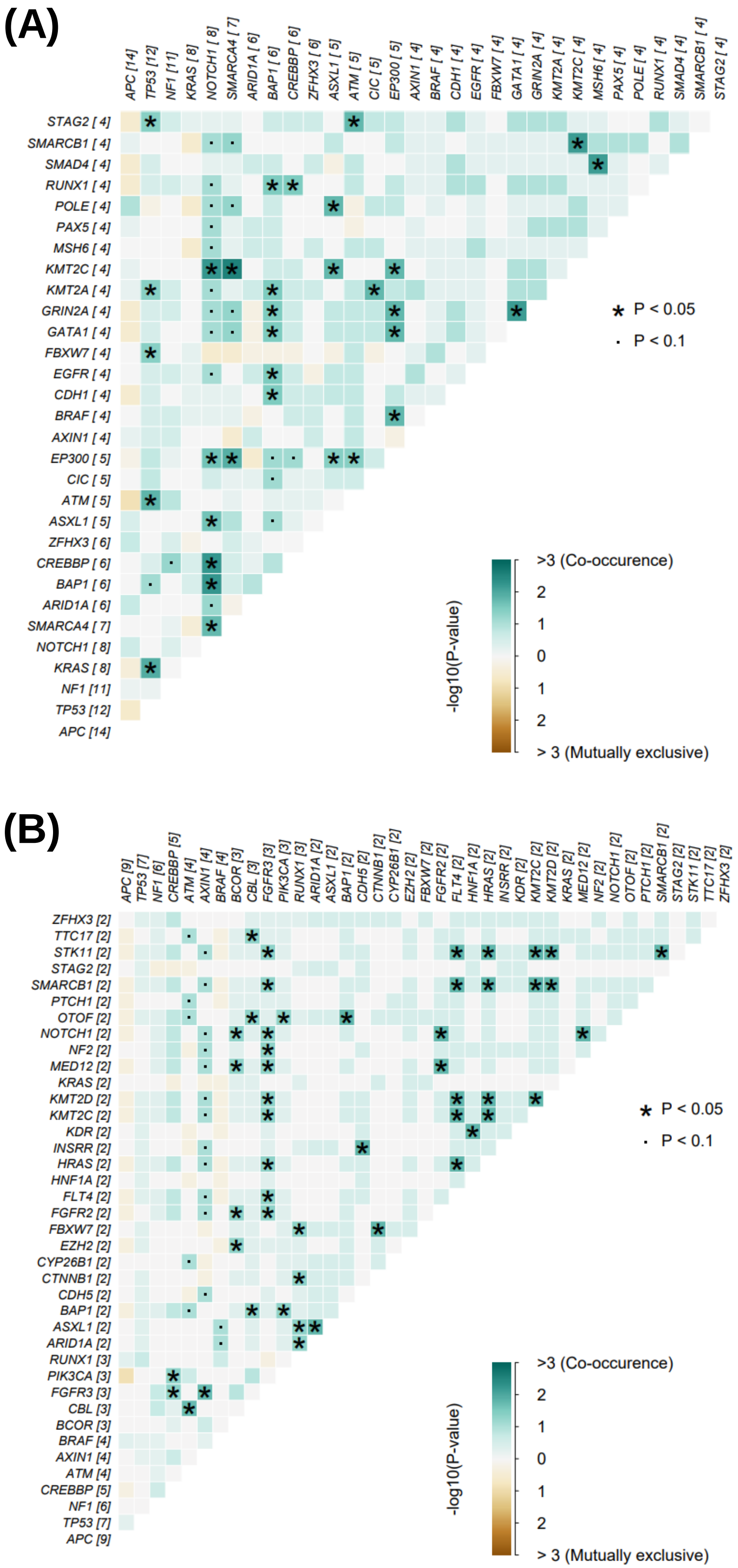

Supplement: Supplementary file 2 — Figure S2. [file CAM4-12-15664-s007.pdf]
