# Supplementary material for: Genomic landscape of locally advanced rectal adenocarcinoma: Comparison between before and after neoadjuvant chemoradiation and effects of genetic biomarkers on clinical outcomes and tumor response
Source: Cancer Med. 2023 Jun 1;12(14):15664–75. doi: 10.1002/cam4.6169 (PMC10417181; doi:10.1002/cam4.6169)
Supplement: Supplementary file 3 — Figure S3. [file CAM4-12-15664-s005.pdf]

Supplementary Figure 3. Kaplan-Meier curves of clinical outcomes significantly associated with gene mutations in pre-chemoradiation samples.

(A) Locoregional control rate

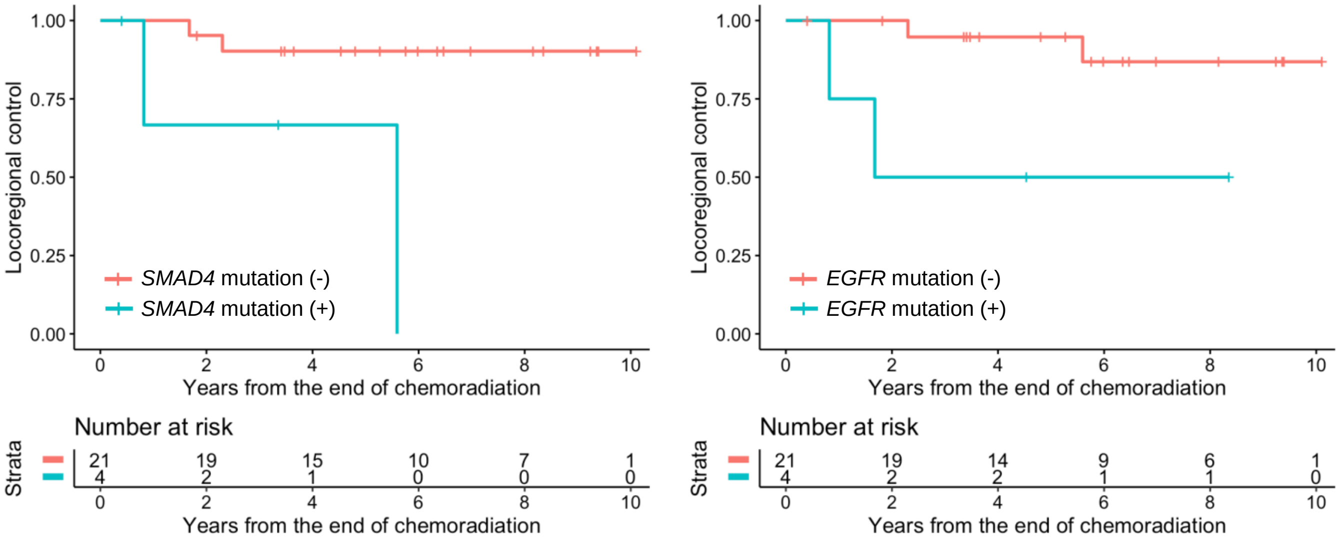

SMAD4
HR 9.571, 95% CI 1.335-68.60, *P* = 0.025

EGFR
HR 7.853, 95% CI 1.077-57.29, *P* = 0.042

(B) Distant metastasis-free rate

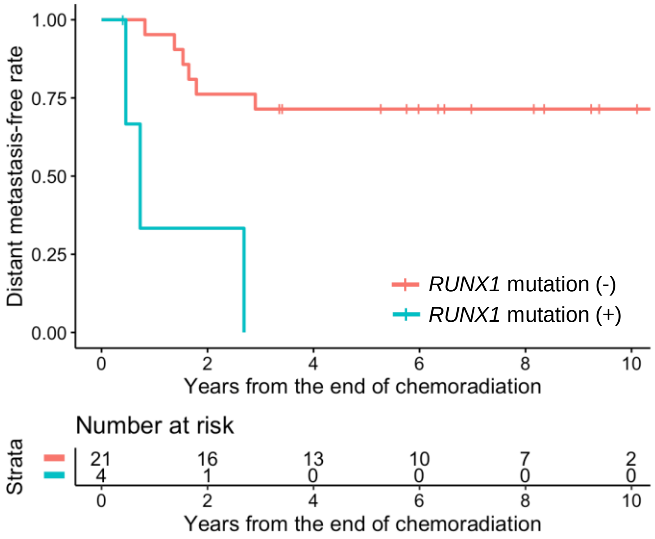

RUNX1
HR 9.023, 95% CI 2.108-38.62, *P* = 0.003

Abbreviations: CI, confidence interval; HR, hazard ratio.

(C) Progression-free survival rate

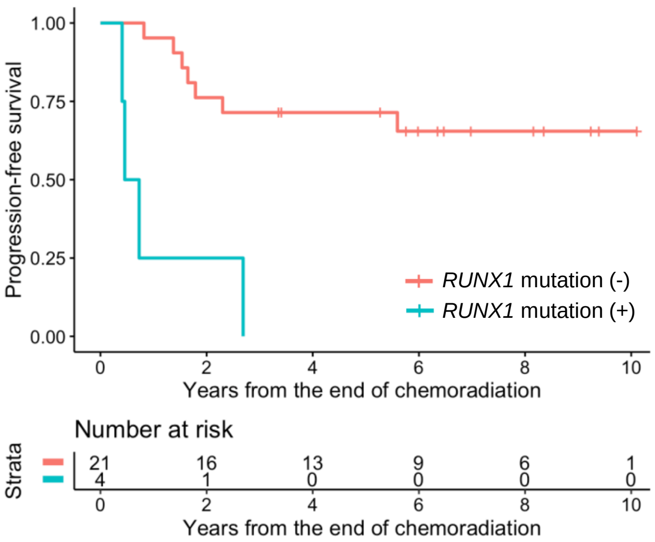

RUNX1
HR 8.993, 95% CI 2.457-32.91, *P* = 0.001

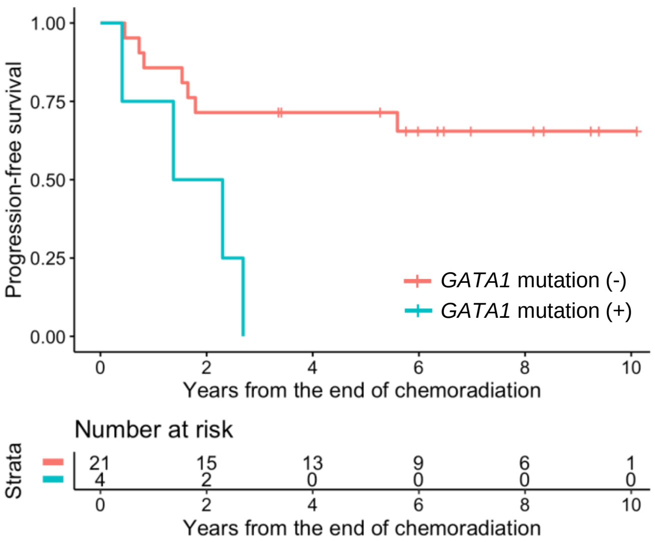

GATA1
HR 5.019, 95% CI 1.402-17.97, *P* = 0.013

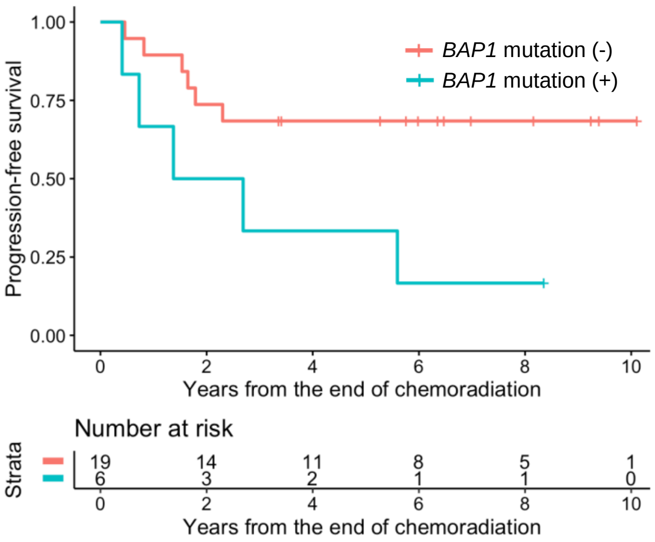

BAP1
HR 3.681, 95% CI 1.119-12.11, *P* = 0.032

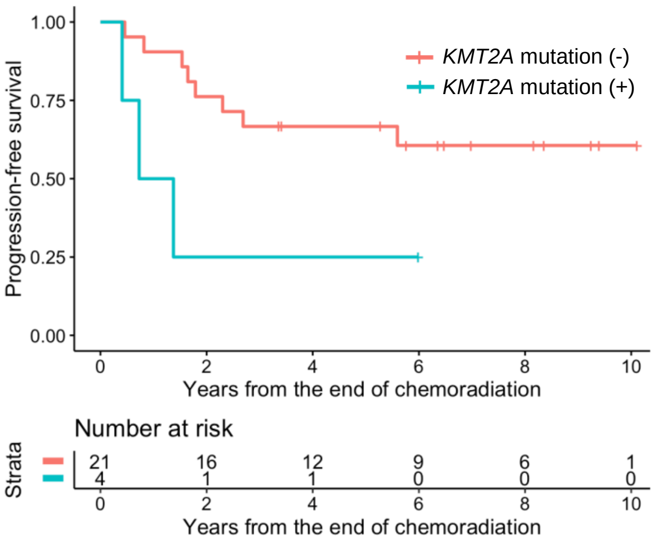

KMT2A
HR 4.045, 95% CI 1.047-15.63, *P* = 0.043

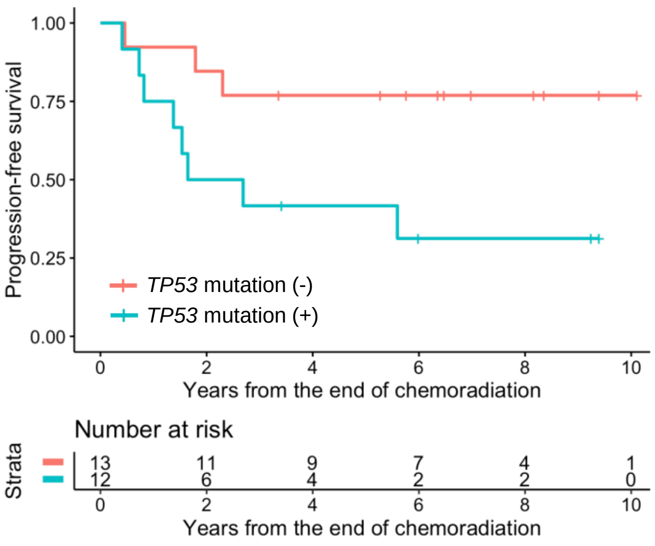

TP53
HR 3.971, 95% CI 1.046-15.07, *P* = 0.043

(D) Overall survival rate

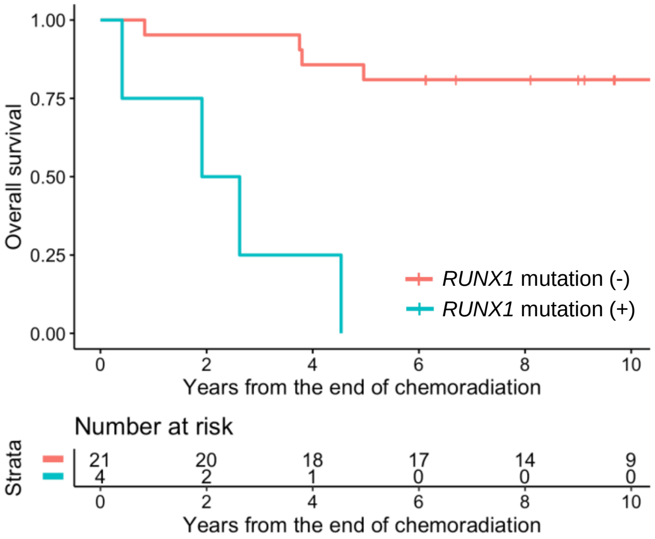

RUNX1
HR 14.64, 95% CI 3.118-68.72, *P* = 0.001

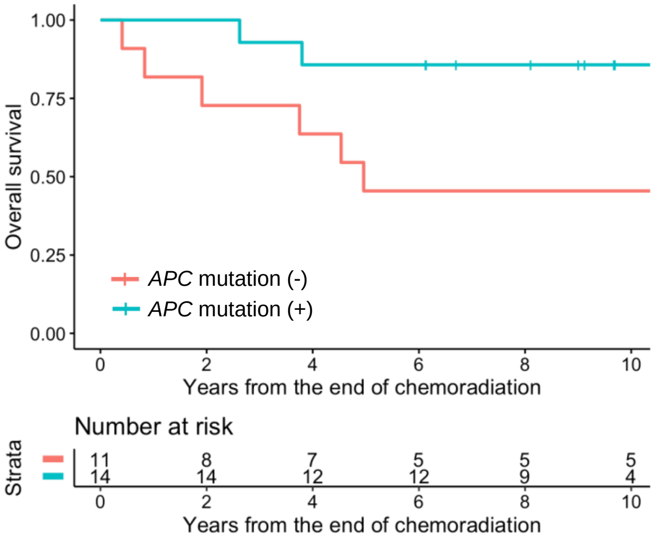

APC
HR 0.187, 95% CI 0.039-0.907, *P* = 0.038

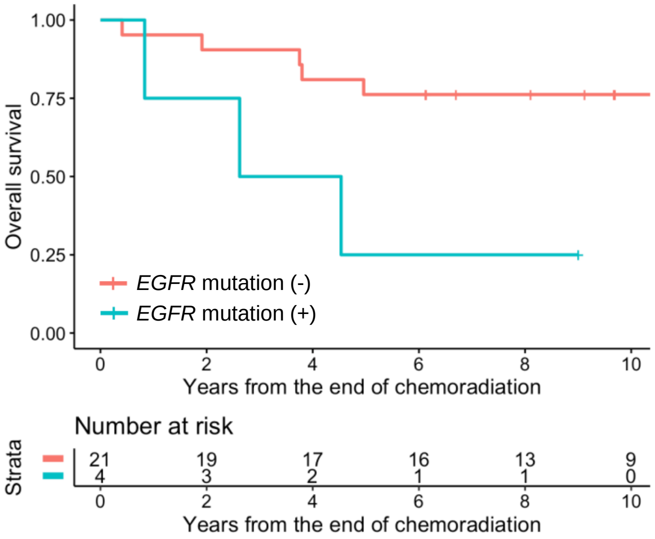

EGFR
HR 4.591, 95% CI 1.075-19.61, *P* = 0.038

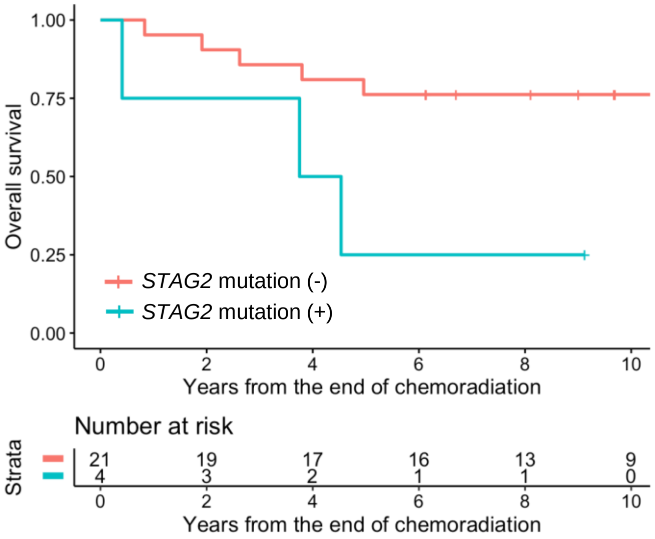

STAG2
HR 4.528, 95% CI 1.064-19.27, *P* = 0.041

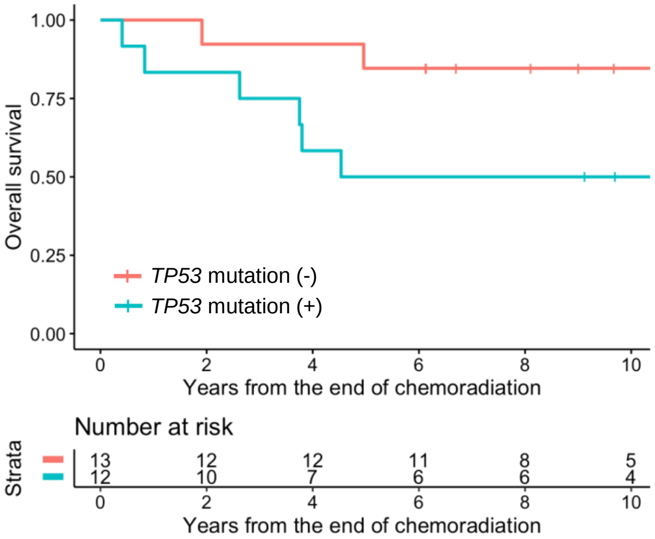

TP53
HR 4.878, 95% CI 1.008-23.59, *P* = 0.049
