# Supplementary material for: Genomic landscape of locally advanced rectal adenocarcinoma: Comparison between before and after neoadjuvant chemoradiation and effects of genetic biomarkers on clinical outcomes and tumor response
Source: Cancer Med. 2023 Jun 1;12(14):15664–75. doi: 10.1002/cam4.6169 (PMC10417181; doi:10.1002/cam4.6169)

Supplementary Figure 4. (A) Relative compositions of doublet-base substitution sorted by DBS1, and (B) Relative compositions of indel signatures sorted by ID6 in pre- and post-chemoradiation samples.

(A) DBS signatures

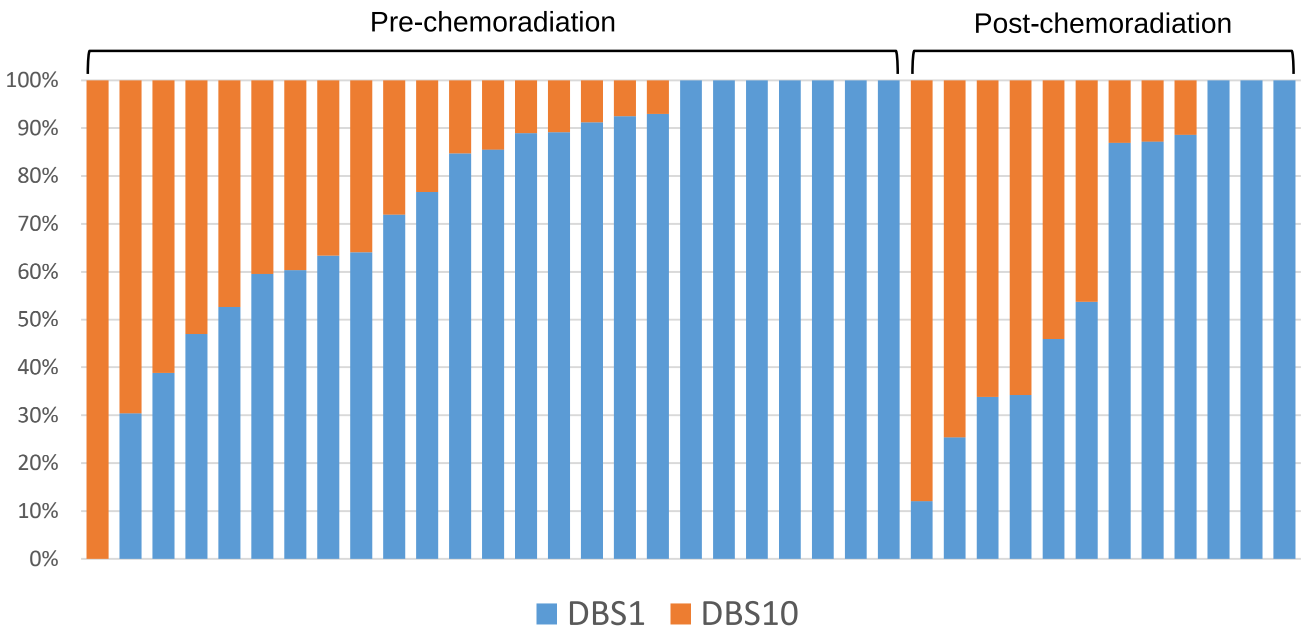

(B) Indel signatures

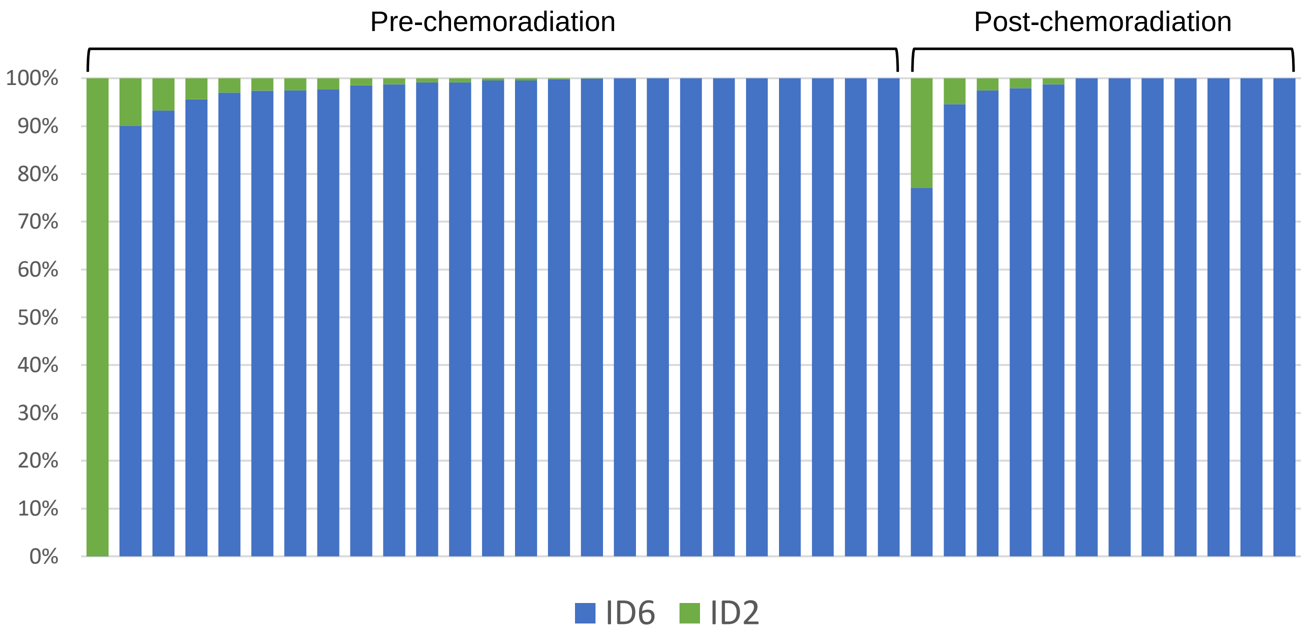

Supplement: Supplementary file 4 — Figure S4. [file CAM4-12-15664-s008.pdf]
