# Supplementary material for: Genomic landscape of locally advanced rectal adenocarcinoma: Comparison between before and after neoadjuvant chemoradiation and effects of genetic biomarkers on clinical outcomes and tumor response
Source: Cancer Med. 2023 Jun 1;12(14):15664–75. doi: 10.1002/cam4.6169 (PMC10417181; doi:10.1002/cam4.6169)

Supplementary Figure 5. Scatter plots for number of (A) single nucleotide variants (SNVs) and (B) indels by pre- and post-chemoradiation (CRT) samples, and box plots for number of SNVs and indels by tumor regression grade in (C) pre- and (D) post-CRT samples.

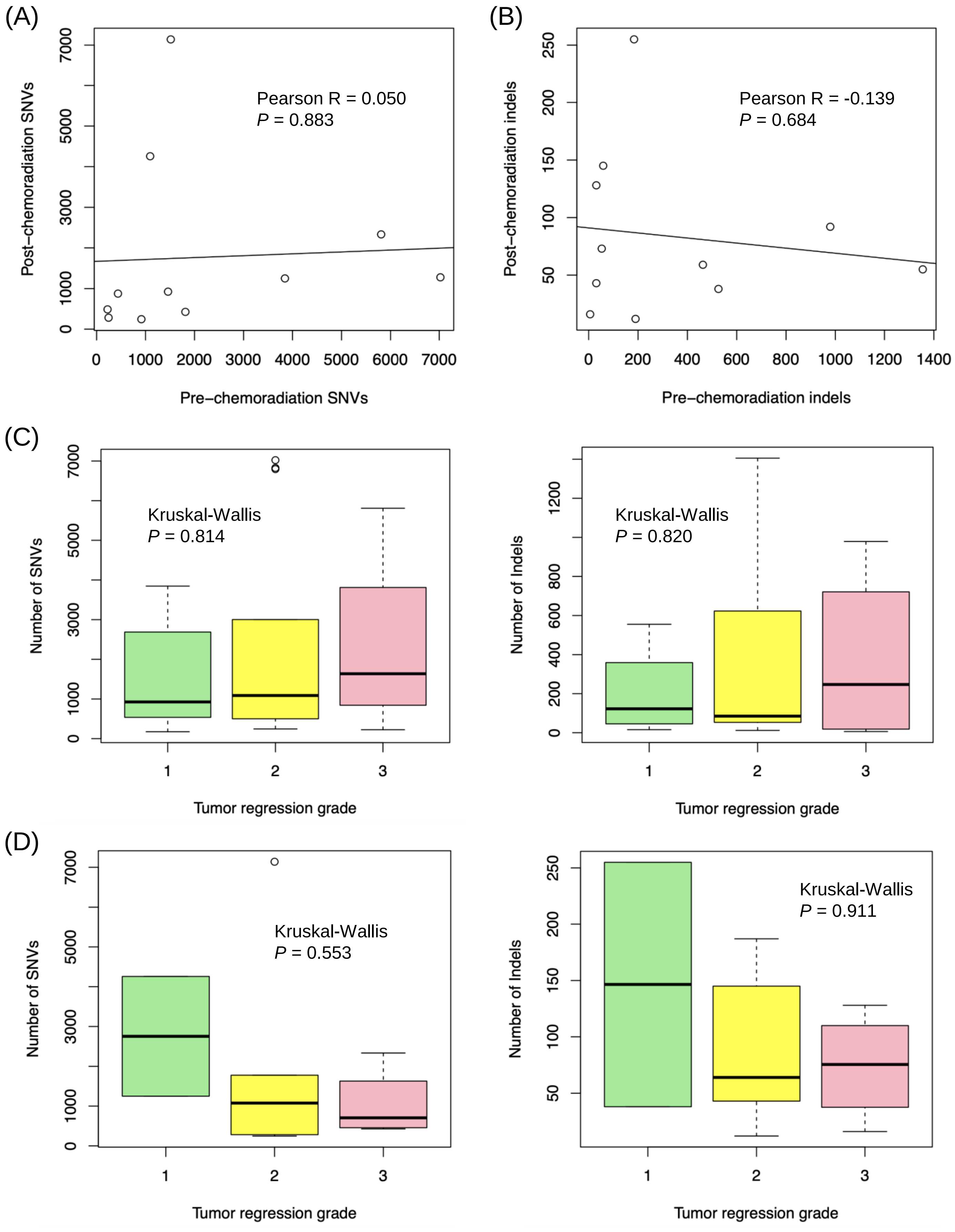

Supplement: Supplementary file 5 — Figure S5. [file CAM4-12-15664-s004.pdf]
