# Supplementary material for: Genomic landscape of locally advanced rectal adenocarcinoma: Comparison between before and after neoadjuvant chemoradiation and effects of genetic biomarkers on clinical outcomes and tumor response
Source: Cancer Med. 2023 Jun 1;12(14):15664–75. doi: 10.1002/cam4.6169 (PMC10417181; doi:10.1002/cam4.6169)

Supplementary Figure 6. (A) Box plots for MATH score by tumor regression grade, (B) Scatter plots for MATH score by pre- and post-chemoradiation samples.

(A)

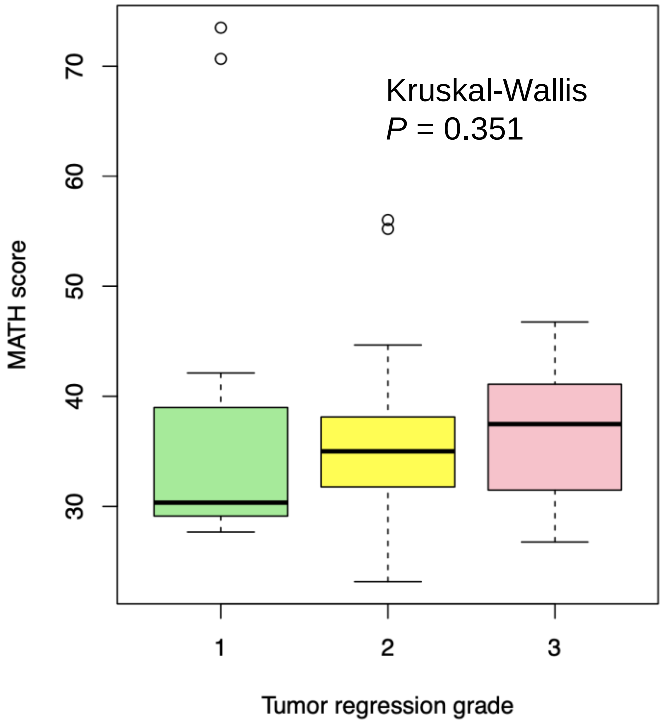

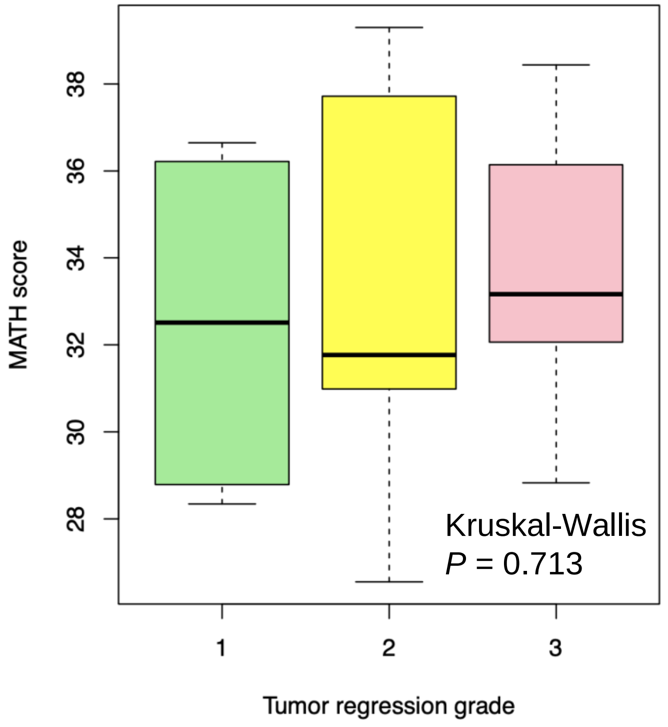

(B)

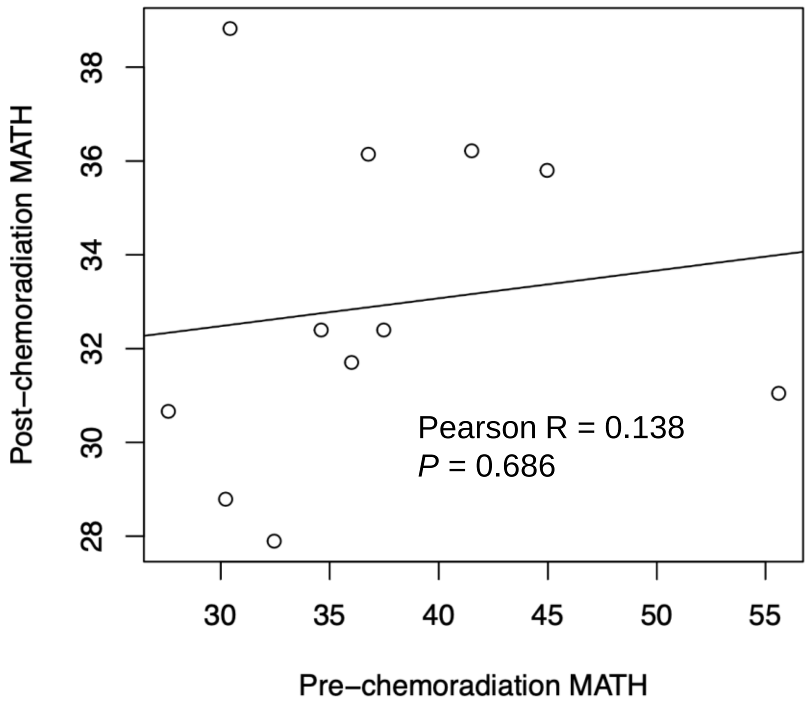

Supplement: Supplementary file 6 — Figure S6. [file CAM4-12-15664-s003.pdf]
