# Supplementary material for: Genomic landscape of locally advanced rectal adenocarcinoma: Comparison between before and after neoadjuvant chemoradiation and effects of genetic biomarkers on clinical outcomes and tumor response
Source: Cancer Med. 2023 Jun 1;12(14):15664–75. doi: 10.1002/cam4.6169 (PMC10417181; doi:10.1002/cam4.6169)
Supplement: Supplementary file 7 — Table S1. [file CAM4-12-15664-s009.docx]

**Supplementary Table 1. Associations of tumor mutational burden with clinical outcomes**

|  | Hazard ratio  (per 100 SNVs / indels) | 95% confidence interval | *P*-value |
| --- | --- | --- | --- |
| **Pre-chemoradiation** |  |  |  |
| Number of SNVs |  |  |  |
| Locoregional control rate | 1.025 | 0.984-1.067 | 0.244 |
| Distant metastasis-free rate | 1.025 | 0.997-1.054 | 0.081 |
| Progression-free survival rate | 1.029 | 1.004-1.054 | 0.024 |
| Overall survival rate | 1.019 | 0.992-1.047 | 0.180 |
| Number of indels |  |  |  |
| Locoregional control rate | 1.152 | 0.953-1.393 | 0.144 |
| Distant metastasis-free rate | 1.142 | 1.009-1.294 | 0.036 |
| Progression-free survival rate | 1.155 | 1.035-1.289 | 0.010 |
| Overall survival rate | 1.117 | 0.987-1.265 | 0.079 |
|  |  |  |  |
| **Post-chemoradiation** |  |  |  |
| Number of SNVs |  |  |  |
| Locoregional control rate | 0.759 | 0.359-1.606 | 0.471 |
| Distant metastasis-free rate | 0.931 | 0.799-1.084 | 0.357 |
| Progression-free survival rate | 0.969 | 0.900-1.044 | 0.413 |
| Overall survival rate | 0.964 | 0.876-1.060 | 0.446 |
| Number of indels |  |  |  |
| Locoregional control rate | 0.439 | 0.010-18.73 | 0.668 |
| Distant metastasis-free rate | 0.433 | 0.076-2.456 | 0.345 |
| Progression-free survival rate | 0.521 | 0.121-2.230 | 0.380 |
| Overall survival rate | 0.554 | 0.109-2.827 | 0.477 |

Cox proportional hazards model was used for calculation.

Abbreviation: SNV, single nucleotide variation.
