# Supplementary material for: Genomic landscape of locally advanced rectal adenocarcinoma: Comparison between before and after neoadjuvant chemoradiation and effects of genetic biomarkers on clinical outcomes and tumor response
Source: Cancer Med. 2023 Jun 1;12(14):15664–75. doi: 10.1002/cam4.6169 (PMC10417181; doi:10.1002/cam4.6169)
Supplement: Supplementary file 8 — Table S2. [file CAM4-12-15664-s002.docx]

**Supplementary Table 2. Estimated sample purity and ploidy**

| **Patient Number** | **Sample Number** | **Estimated Sample Purity** | **Estimated Ploidy** |
| --- | --- | --- | --- |
| **Pre-chemoradiation** | | | |
| 1 | 1 | 0.40 | 1.8 |
| 1 | 2 | 0.51 | 1.9 |
| 2 | 1 | 0.28 | 6.4 |
| 2 | 2 | 0.25 | 2.4 |
| 5 | 1 | 0.41 | 1.8 |
| 5 | 2 | 0.43 | 1.8 |
| 8 | 1 | 0.66 | 2.0 |
| 8 | 2 | 0.65 | 2.0 |
| 12 | 1 | 0.10 | 1.3 |
| 12 | 2 | 0.09 | 1.2 |
| 14 | 1 | 0.26 | 6.1 |
| 14 | 2 | 0.25 | 6.4 |
| 15 | 1 | 0.17 | 1.8 |
| 15 | 2 | 0.12 | 2.0 |
| 16 | 1 | 0.09 | 2.3 |
| 16 | 2 | 0.05 | 2.4 |
| 17 | 1 | 0.22 | 2.0 |
| 17 | 2 | 0.14 | 2.1 |
| 18 | 1 | 0.27 | 5.0 |
| 18 | 2 | 0.30 | 4.8 |
| 19 | 1 | 0.18 | 2.3 |
| 19 | 2 | 0.12 | 2.8 |
| 21 | 1 | 0.15 | 3.1 |
| 21 | 2 | 0.15 | 3.4 |
| 22 | 1 | 0.23 | 4.8 |
| 22 | 2 | 0.25 | 4.9 |
| 23 | 1 | 0.22 | 5.9 |
| 23 | 2 | 0.23 | 5.7 |
| 24 | 1 | 0.30 | 2.1 |
| 24 | 2 | 0.27 | 2.1 |
| 25 | 1 | 0.42 | 2.1 |
| 25 | 2 | 0.42 | 2.2 |
| 26 | 1 | 0.38 | 2.8 |
| 26 | 2 | 0.39 | 2.8 |
| **Post-chemoradiation** | | | |
| 7 | 1 | 0.37 | 2.1 |
| 7 | 2 | 0.38 | 2.4 |
| 10 | 1 | 0.96 | 1.9 |
| 10 | 2 | 0.39 | 1.7 |
| 14 | 1 | 0.51 | 2.9 |
| 14 | 2 | 0.52 | 2.9 |
| 15 | 1 | 0.08 | 2.1 |
| 15 | 2 | 0.75 | 2.0 |
| 16 | 1 | 0.06 | 1.0 |
| 16 | 2 | 0.07 | 1.4 |
| 18 | 1 | 0.08 | 2.3 |
| 18 | 2 | 0.07 | 2.4 |
| 20 | 1 | 0.13 | 2.1 |
| 20 | 2 | 0.16 | 2.2 |
| 22 | 1 | 0.15 | 4.7 |
| 22 | 2 | 0.14 | 4.5 |
| 23 | 1 | 0.13 | 1.1 |
| 23 | 2 | 0.13 | 1.3 |
| 24 | 1 | 0.19 | 1.9 |
| 24 | 2 | 0.07 | 1.6 |
| 25 | 1 | 0.44 | 2.0 |
| 25 | 2 | 0.45 | 2.0 |
| 26 | 1 | 0.19 | 3.6 |
| 26 | 2 | 0.18 | 3.9 |
