# Supplementary material for: Genomic landscape of locally advanced rectal adenocarcinoma: Comparison between before and after neoadjuvant chemoradiation and effects of genetic biomarkers on clinical outcomes and tumor response
Source: Cancer Med. 2023 Jun 1;12(14):15664–75. doi: 10.1002/cam4.6169 (PMC10417181; doi:10.1002/cam4.6169)
Supplement: Supplementary file 9 — Table S3. [file CAM4-12-15664-s001.docx]

**Supplementary Table 3. List of GISTIC2 amplification and deletion peaks in pre- and post-chemoradiation samples**

| **Type** | **Descriptor** | **Wide Peak Limits** | **Q-value** |
| --- | --- | --- | --- |
| **Pre-chemoradiation** | | | |
| Amplification | 1q21.3 | chr1:152214708-152358763 | 0.014 |
| Amplification | 4p11 | chr4:48561089-52917612 | 0.002 |
| Amplification | 6p21.1 | chr6:41584796-43443364 | 0.001 |
| Amplification | 7q22.1 | chr7:100954062-100956479 | 0.003 |
| Amplification | 12q12 | chr12:39854856-40489822 | < 0.001 |
| Amplification | 16q11.2 | chr16:34158851-46401173 | 0.002 |
| Amplification | 20q11.1 | chr20:26071629-30815511 | 0.055 |
| Deletion | 2q37.3 | chr2:231493530-242193529 | 0.097 |
| Deletion | 3p12.3 | chr3:75672123-75737023 | < 0.001 |
| Deletion | 4p11 | chr4:1-190214555 | < 0.001 |
| Deletion | 4p11 | chr4:48551167-52907613 | < 0.001 |
| Deletion | 5q11.2 | chr5:49651846-52871266 | < 0.001 |
| Deletion | 6p21.32 | chr6:32589734-32741592 | 0.093 |
| Deletion | 7p11.2 | chr7:56077388-63629923 | 0.085 |
| Deletion | 7q22.1 | chr7:100810077-100969663 | < 0.001 |
| Deletion | 8p23.1 | chr8:11769691-12455364 | 0.097 |
| Deletion | 9q13 | chr9:42784720-62801650 | 0.043 |
| Deletion | 12q12 | chr12:1-133275309 | 0.085 |
| Deletion | 12q12 | chr12:34209511-40488161 | 0.085 |
| Deletion | 13q11 | chr13:18230172-19178748 | 0.027 |
| Deletion | 14q11.2 | chr14:1-18994699 | 0.010 |
| Deletion | 14q32.33 | chr14:106284978-107043718 | 0.046 |
| Deletion | 15q11.2 | chr15:1-23575742 | 0.097 |
| Deletion | 16q11.2 | chr16:34158851-48200415 | 0.001 |
| Deletion | 17p11.2 | chr17:21545188-27560503 | 0.085 |
| Deletion | 19p12 | chr19:1-58617616 | 0.085 |
| Deletion | 19p12 | chr19:21163934-29613828 | 0.085 |
| Deletion | 20q11.1 | chr20:26061756-32216519 | 0.027 |
| **Post-chemoradiation** | | | |
| Amplification | 3p12.3 | chr3:75665273-75738424 | 0.023 |
| Amplification | 6p21.1 | chr6:41584802-43350622 | 0.045 |
| Amplification | 11q13.3 | chr11:69077921-71557391 | 0.001 |
| Amplification | 13q11 | chr13:1-18273528 | 0.001 |
| Amplification | 20q11.1 | chr20:29678346-29800133 | 0.005 |
| Deletion | 4p11 | chr4:48906358-52597557 | < 0.001 |
| Deletion | 16q11.2 | chr16:35473236-48200415 | < 0.001 |
| Deletion | 17q11.1 | chr17:1-83257441 | < 0.001 |
| Deletion | 17q11.1 | chr17:21703502-27301793 | < 0.001 |
